# Synthesis and Characterization of Preacinetobactin and 5-Phenyl Preacinetobactin

**DOI:** 10.3390/molecules27123688

**Published:** 2022-06-09

**Authors:** Jean M. Bray, Scott Pierce, Alfredo M. Angeles-Boza, Mark W. Peczuh

**Affiliations:** Department of Chemistry, University of Connecticut, 55 N. Eagleville Road, U3060, Storrs, CT 06269, USA; jean.bray@uconn.edu (J.M.B.); scott.pierce@uconn.edu (S.P.); alfredo.angeles-boza@uconn.edu (A.M.A.-B.)

**Keywords:** acinetobactin, preacinetobactin, siderophore, synthesis, *A. baumanii*

## Abstract

We report the first total synthesis of 5-phenyl preacinetobactin and its characterization. The route was developed for the synthesis of preacinetobactin, the siderophore critical to the Gram-negative pathogen *A. baumannii*. It leverages a C5-substituted benzaldehyde as a key starting material and should enable the synthesis of similar analogs. 5-Phenyl preacinetobactin binds iron in a manner analogous to the natural siderophore, but it did not rescue growth in a strain of *A. baumannii* unable to produce preacinetobactin.

## 1. Introduction

Selection pressures have facilitated the appearance of *Acinetobacter baumannii* strains in hospital settings that are either multi-drug resistant (MDR) or extensively drug resistant (XDR), with mortality rates up to 70% for XDR strains [1,2]. In the last couple of years both the World Health Organization and the Centers for Disease Control have marked carbapenem-resistant A. *baumannii* at the highest threat level [3,4]. The rapid ascendance of XDR strains demands new treatment methods for *A. baumannii* infections [5,6]. A significant challenge to the development of new treatments, however, is the impermeability of the cell envelope of this Gram-negative organism [7,8]. New ways to bypass the cell membrane could suggest strategies for promising new treatments for XDR *A. baumannii*. One such strategy is the Trojan Horse approach [9,10]. Just as in the story of the Greek army that smuggled their soldiers into the city of Troy, molecular cargo such as an antibiotic, can be smuggled through the cell envelope of Gram-negative bacteria by attaching it to the native substrate of a transporter protein. This approach hijacks the natural active transport system to deliver an antibiotic into the cell by linking a small molecule native to the bacterial target to an antibacterial warhead. In fact, the strategy is one utilized in Nature; sideromycins are a class of natural Trojan Horse antibiotics that utilize the siderophore transport machinery to deliver cytotoxins inside bacterial cells [11].

**Figure 1:**
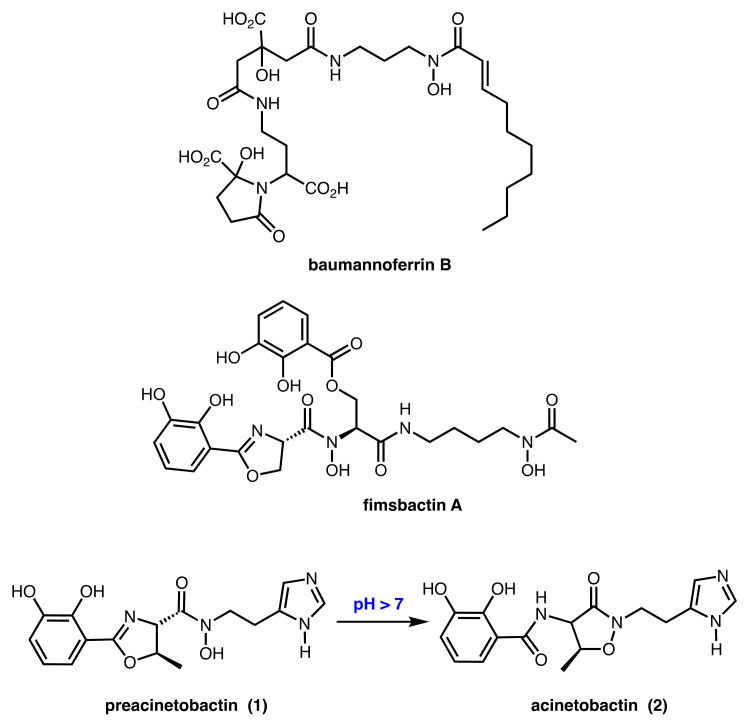
Siderophores produced and used by *A. baumannii:* baumannoferrin B (**top**), fimsbactin A (**middle**), and pre/acinetobactin (**bottom**). Preacinetobactin **1** isomerizes to acinetobactin **2** at pH > 7.

*A. baumannii* is particularly resilient to many environments, but the acquisition of iron is still required for both its survival and pathogenicity [12,13]. Host organisms sequester iron to maintain a very low concentrations of free iron, creating a problem for bacterial iron acquisition. In order to counter the low-iron environment created by the host, Gram-negative bacteria utilize siderophores to obtain iron. Siderophores are essential small molecules that chelate iron and have antibiotic conjugates that have been studied for their antibiotic activity [14,15,16,17,18], and some specifically for *A. baumannii* [19,20]. *A. baumannii* is known to produce three siderophore types: baumannoferrin, fimsbactin, and pre/acinetobactin (Figure 1) [21]. Recent work has shown that while all three siderophores are produced in many strains of *A. baumannii,* during iron-restriction, pre/acinetobactin is the only siderophore essential to its survival [22].

Nonetheless, siderophore-antibiotic conjugates targeting *A. baumannii* have thus far only focused on fimsbactin analogs [19,20]. Although fimsbactins A-F have been observed in many clinical isolates, pre/acinetobactin remains the primary siderophore that is highly conserved across *A. baumannii* strains, highlighting it as the ideal target for a siderophore-antibiotic conjugate [23]. Pre/acinetobactin exists as two isomers, preacinetobactin and acinetobactin (Figure 1). Preacinetobactin is biosynthesized and spontaneously isomerizes to acinetobactin as a result of an increase in pH above pH 7. It has been argued that both isomers exist because they broaden the pH range over which iron can be sequestered [24]. On the other hand, a report on the crystal structure of the BauA transporter (vide infra) shows that only preacinetobactin can initiate active transport [25]. Pre/acinetobactin binds with Fe(III) in an uncommon 2:1 stoichiometry, in contrast to baumannoferrin and fimsbactin which bind in a 1:1 siderophore:Fe(III) relationship. The unusual features of acinetobactin/preacinetobactin, combined with the importance of the small molecule, identify it as a desirable candidate for a Trojan Horse antibiotic. This work developed a modified total synthesis of preacinetobactin that is amenable to derivatization for the goal of a preacinetobactin-antibiotic conjugate.

## 2. Results

### 2.1. Planning and Design

**Figure 2:**
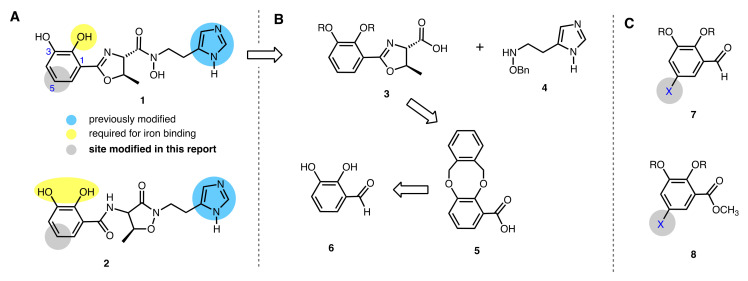
(**A**) Sites for modification of preacinetobactin **1** and acinetobactin **2**. (**B**) Retrosynthesis of preacinetobactin **1** based on the new synthetic route. (**C**) Starting materials **7** and **8** envisioned for C5-functionalized preacinetobactin analogs.

Design considerations that motivated the new siderophore conjugates grew out of the structures of co-crystals of pre/acinetobactin complexed to proteins involved in the siderophore transport pathway that have been reported recently [25,26]. *A. baumannii* uses a TonB dependent outer membrane protein, BauA, to transport iron bound siderophores from outside the cell into the periplasm. Once in the periplasm, the siderophore complexes are bound to a periplasmic siderophore-binding protein, BauB, that facilitates transport through an ABC transporter (BauCDE) into the cytoplasm [24]. BauA, crystalized with a preacinetobactin:acinetobactin heterodimer bound to iron, revealed a potential site of conjugation at C5 (Figure 2A) [25]. The BauB crystal is contained an iron-bound acinetobactin dimer; this complex retains the availability for C5 conjugation [26].

While the hetero vs. homo dimer is conflicting in some respects, the two crystal structures of BauA and BauB confirm that the C5 position is the most accessible to functionalization and, therefore, most likely to allow transport into the cytoplasm for a siderophore-antibiotic conjugate. Pre/acinetobactin analogs have also been made to investigate the important structural considerations of the siderophore by Kim et al. [27] and Wencewicz et al. [28]. As shown by Wencewicz, a 2,5-OH preacinetobactin was tolerated by *A. baumannii* and retained iron binding and siderophore activity. Together, data from the co-crystal structures and analog studies suggest that C5 is a promising position for conjugation.

Inspection of the structure of preacinetobactin **1** pointed to a clear retrosynthetic disconnection at the substituted carbamic acid unit (Figure 2B). This disconnection revealed a catechol-functionalized oxazoline carboxylic acid (**3**) and a (imidazolyl-ethyl) hydroxylamine (**4**). Previous reports utilized this same disconnection and established complementary synthetic routes to intermediates that could then participate in the key condensation step that units them. Reaction of an aromatic imidate with l-threonine led to oxazoline carboxylic acid **3** in the approach described by Takeuchi et al. [29], whereas Kim et al. prepared it by way of an aromatic l-threonine amide cyclization [30]. Access to (imidazolyl-ethyl) hydroxylamine **4** was either via oxidation of histamine or de novo synthesis by cyclization with an alpha hydroxy ketone and formaldehyde. We opted to prepare **3** starting from a catecholic aldehyde (i.e., **6**) that could be protected and oxidized to carboxylic acid **5**, then elaborated to **3** using the Kim amidation-cyclization strategy. The strategy we introduce here, therefore, constitutes a new synthetic route that showcases an alternative and versatile approach to **3**. Its merit resides in the facile ability to introduce functionality at the C5 position via catecholic aldehyde starting materials, such as **7,** instead of esters such as **8** (Figure 2C). Here we report a new synthetic route to preacinetobactin that works as a scaffold for the development of a 5-phenyl analog of preacinetobactin.

### 2.2. Synthesis of Preacinetobactin

**Scheme 1:**
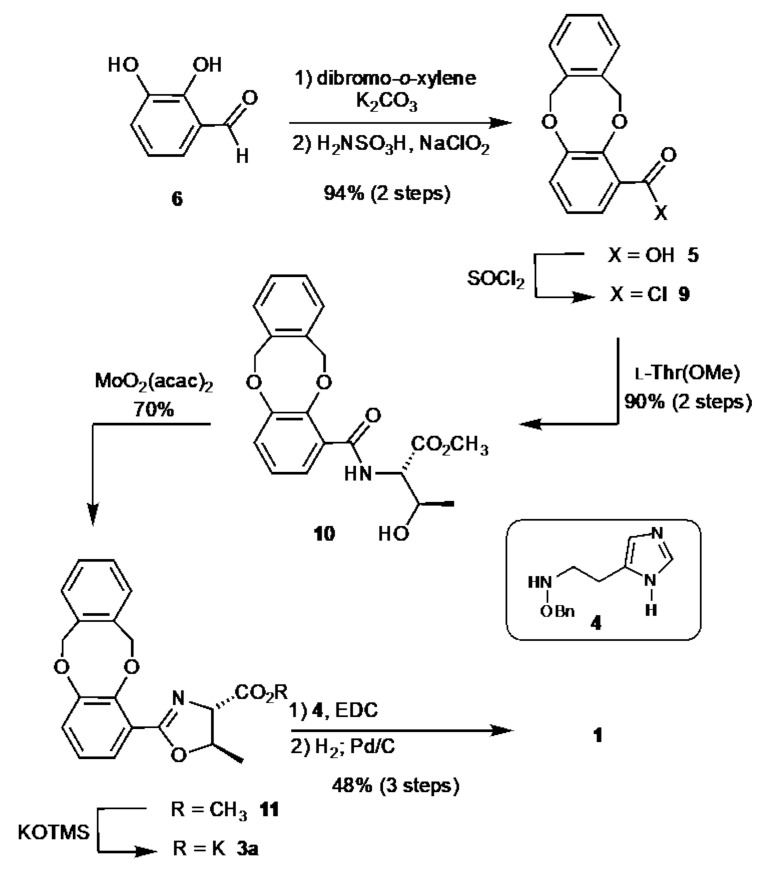
Synthesis of preacinetobactin **1**.

Synthesis of preacinetobactin **1** by the new route is depicted in Scheme 1. Conversion of 2,3-dihydroxy benzaldehyde **6** to oxazoline carboxylate **3a** over six steps constitutes the first phase of the synthesis. Protection of the catechol moiety of **6** as its xylylene ether initiated the sequence and was followed by the key Pinnick oxidation, giving rise to carboxylic acid **5** in 94% yield over the two steps. Formation of l-threonine amide **10** was then affected by conversion of **5** to its corresponding acid chloride, **9**, followed by attachment of an l-threonine methyl ester in 90% yield. Molybdenum oxide-catalyzed dehydrative cyclization then converted amide **10** into oxazoline **11** in 70% yield. Conversion of the methyl ester moiety of **11** to its potassium carboxylate salt through the action of KOTMS gave key intermediate **3a**.

With **3a** in hand, the end-phase of the synthesis leveraged EDC-mediated condensation with benzyl-protected (imidazolyl-ethyl) hydroxyamine **4**, prepared via Kim’s alpha hydroxy ketone cyclization strategy, to produce protected preacinetobactin in 49% yield. Hydrogenolysis of the xylylene and benzyl protecting groups then provided preacinetobactin **1** (97%). Evaluation of the new synthetic route shows that the product is obtained in 28% yield over eight synthetic steps and >70% average yield per step. It is worth noting that there are three two-step operations that facilitate through-put in the synthesis. Our route is comparable to the route by Kim et al., which reported a 33% yield over eight steps. With many similarities between our routes, the key difference is in accessing carboxylic acid **5**. Our Pinnick oxidation strategy allows access to **5** in 97% yield, while the ester route has a 67% yield. Both are more efficient than the 6% overall yield (six steps) reported by Takeuchi et al. as the first known route.

### 2.3. Synthesis of 5-phenyl Preacinetobactin

The route established for preacinetobactin **1** was then leveraged toward the synthesis of a derivative containing a phenyl group at the C5 position of its catecholic ring.

**Scheme 2:**
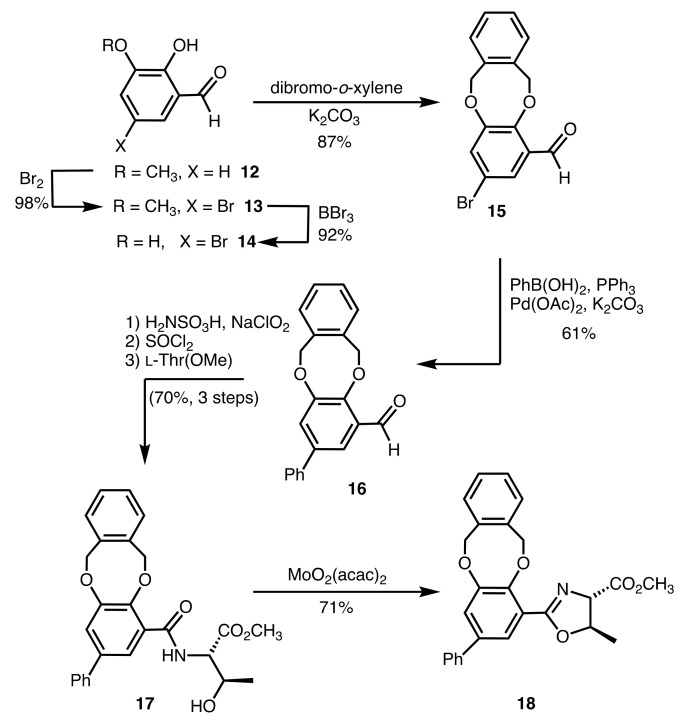
Synthesis of C5-functionalized oxazoline **18**.

**Scheme 3:**
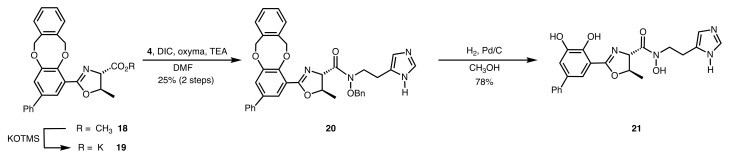
Synthesis of 5-phenyl preacinetobactin **21**.

Our approach hinged on ready access to aldehyde starting materials and the ability to introduce functionality on the aromatic ring at this oxidation state relative to the carboxylic acid. We turned to *o*-vanillin **12** as a starting material as a latent analog of 2,3-dihydroxy benzalde-hyde **6**. The C5 position of **12** can be selectively derivatized via substitution chemistry. For example, bromination of **12** resulted in the regioselective installation of bromine at C5, producing **13** in 98% yield (Scheme 2). Demethylation of **13** with BBr_3_ gave 5-bromo-2,3-dihydroxy benzaldehyde **14** (92%) whose catechol unit was then protected as the xylylenyl ether **15** (87%). Suzuki coupling between aldehyde **15** and phenyl boronic acid produced 5-phenyl benzaldehyde **16** in 61% yield. Early on, we attempted to elaborate the C5 aryl bromide at a later stage in the synthesis. These attempts included protected 5-bromo-precinetobactin and a 5-bromo oxazoline methyl ester (akin to **11**), but these reactions were not successful (Schemes S1 and S2). Once in hand, benzaldehyde **16** was subjected to the same set of reactions that were used previously to prepare preacinetobactin. Pinnick oxidation converted the aldehyde to the carboxylic acid, which was carried to l-threonine amide **17** (70% over three steps). Formation of the oxazoline moiety by molybdenum catalyzed cyclization delivered methyl ester **18** in 71% yield. Cleavage of the methyl ester to form potassium carboxylate **19** (Scheme 3) was followed by condensation with benzyl-protected (imidazolyl-ethyl) hydroxyamine **4** using DIC and oxyma as coupling reagents to provide protected 5-phenyl preacinetobactin **20** (25% over two steps). Use of the DIC-oxyma pair gave higher yields than other reagents such as EDC and HBTU for this coupling. Hydrogenolysis then provided access to 5-phenyl preacinetobactin **21** in 78% yield.

### 2.4. Characterization by Metal Binding and Growth Recovery Assays

**Figure 3:**
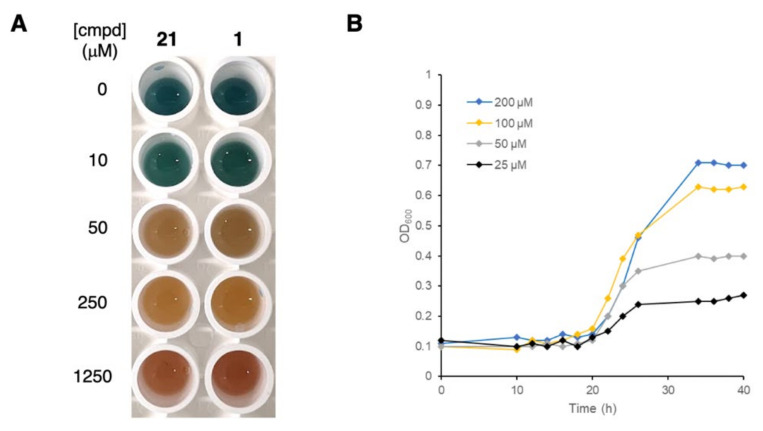
Fe(III) binding characterization of **1** and **21**. (**A**) Results of CAS Fe(III) binding assay with compounds **1** and **21**. (**B**) Growth recovery assay of **1** with ATCC 19606-s1.

We next sought to characterize the iron binding capabilities of preacinetobactin **1** and the new 5-phenyl-preacinetobactin **21**. Colorimetric evaluation of Fe(III) binding was conducted by the Chrome Azurol S (CAS) assay. Solutions of **1** and **21** resulted in a change in color from blue to orange indicating iron binding (Figure 3, Table S1 and Figure S1). The affinity of preacinetobactin **1** and 5-phenyl preacinetobactin **21** for Fe(III) was also measured by monitoring the change in optical absorbance of each respective siderophore as the concentration of tris(acetylacetonato) iron(III) was varied. Changes in absorbance at 650 nm of **1** were comparable to those reported previously [24] and confirmed a 2:1 siderophore:iron binding stoichiometry (Figures S2 and S3). A red shift in the UV-vis spectra over time was interpreted as a consequence of the isomerization of the preacinetobactin to acinetobactin (Figure 1), as could be expected from the length of time between Fe(III) addition and UV-vis measurement [24]. UV-Vis titration of **21** was done in DMSO due to the insolubility of the Fe(III):5-phenyl preacinetobactin complex in water. The optical absorbance of **21** showed similar behavior to **1**, which provided evidence that the derivatization of C5 had not compromised its ability to bind Fe(III).

Growth recovery assays using *A. baumannii* ATCC 19606-s1 were also employed to characterize the siderophore activity of preaceinetobactin **1** from our synthetic route and 5-phenyl preacinetobactin **21** [27,28] *A. baumannii* ATCC 19606-s1 is a *ba*s*D* insertional mutant that disrupts the biosynthesis of preacinetobactin but retains all genes related to siderophore transport [31]. The growth recovery of *A. baumannii* ATCC 19606-s1 in the presence of **1** further demonstrated the activity of the newly synthesized preacinetobactin. Cultures of *A. baumannii* ATCC 19606-s1 supplemented with **1** showed recovery of growth in a dose-dependent manner (Figure 3) in line with previous reports on its activity. Growth recovery was not observed, however, when 5-phenyl preacinetobactin **21** was added to *A. baumannii* ATCC 19606-s1 cultures over the range of concentrations that were tested (Figure S4). The lack of growth recovery could depend on multiple factors. First, the substitution at C5 of **21** may, in fact, prevent binding to BauA and BauB. Alternatively, binding may occur, but without preacinetobactin **1**, transport may be stopped or severely restricted. It may be that, due to the poor aqueous solubility of the dimer of **21** with Fe(III), there was an insufficient concentration of siderophore available, even in a scenario where C5 groups could be accommodated by these proteins. We plan to investigate this question in future work with other C5 functionalized preacinetobactin analogs.

## 3. Discussion

A new synthetic route for the preparation of preacinetobactin **1** is reported here. The route uses the oxidation of a benzyaldehyde moiety to the corresponding benzoic acid as a key step in the synthesis. This oxidation step increased the yield to intermediate **5** from 67 to 97%, increasing the availability of this key intermediate. Critically, the new route has also enabled modifications of the pre/acinetobactin structure to include C5-modified analogs. As a proof-of-concept, 5-phenyl preacinetobactin **21** was synthesized using the approach. Integral to its application was the ability to prepare a protected 5-bromo-2,3,-*O*-xylenyl-benzaldehyde **15** that then participated in a Suzuki coupling with phenyl boronic acid. 5-Phenyl preacinetobactin **21** was then characterized for its ability to bind iron and act as a siderophore. While iron binding was essentially the same as preacinetobactin **1**, 5-phenyl derivative **21** failed to rescue growth in an *A. baumannii* strain unable to biosynthesize preacinetobactin. The poor solubility of **21** was the most likely factor that prevented rescue. Though **21** was not a functional siderophore, the new synthetic route will be central to the development of other derivatives. We are actively investigating the synthesis and properties of other C5-functionalized preacinetobactin analogs and will report on them in due course.

## 4. Materials and Methods

### 4.1. Synthetic Procedures

*2,3-(o-xylylenyl-di-oxy)benzoic acid* (**5**) 2,3-dihydroxy benzaldehyde (2.00 g, 14.5 mmol) and K_2_CO_3_ (5.90 g, 43.5 mmol) were dissolved in 60 mL DMF in a 250 mL 3-neck round bottom flask and heated to 130 °C. In a separate flask α,α’-dibromo-*o*-xylene (4.50 g, 17.4 mmol) was dissolved in 60 mL DMF and this solution was added dropwise over 20 min to the benzaldehyde and K_2_CO_3_ mixture. The reaction was maintained at 130 °C for 1 hr and then the mixture was allowed to cool to rt. The mixture was diluted with 400 mL water and extracted with Et_2_O (3 × 50 mL). The combined organic layers were washed with 10% aq. NaOH (2 × 25 mL) and brine (1 × 30 mL). The reaction yielded 3.38 g (97%) of a pale yellow/brown solid. The 2,3-(*o*-xylylenyl-di-oxy)benzaldehyde (3.35 g, 13.9 mmol) was dissolved in 14 mL acetone and diluted with 14 mL H_2_O. Sulfamic acid (1.89 g, 19.6 mmol) was added to this solution, followed by portion-wise addition of solid NaClO_2_ (1.26 g, 13.9 mmol). The mixture was stirred for 1 h at rt and was then concentrated under reduced pressure. Ethyl acetate (30 mL) was added, and the organic solution was washed with water (1 × 50 mL). The organic layer was dried over Na_2_SO_4_, filtered and the solvent was removed under reduced pressure to afford the product in quantitative yield (3.55 g) as a white solid. m.p. 99-101 °C; R_f_ 0.23 (40% EtOAC:Hexanes); ^1^H NMR (400 MHz, CDCl_3_) δ 7.88 (dd, J = 7.8,1.7 Hz, 1H), 7.35 (m, 4H), 7.16 (m, 1H), 7.09 (t, J = 7.95 Hz, 1H), 5.74 (s, 2H), 5.41 (s, 2H). ^13^C NMR (100 MHz, CDCl_3_) δ 165.6, 149.8, 149.6, 136.3, 132.5, 130.6, 129.7, 128.9, 128.2, 128.1, 127.8, 123.5, 121.3, 76.7, 75.6; HRMS (ESI-TOF) for C_15_H_13_O_4_ [M+H]^+^ *m*/*z* calcd 257.0808, obsd. 257.0782.

*N-[(2,3-o-xylylenyl-di-oxy)benzoyl]-l-threonine methyl ester* (**10**) Compound **5** (0.332 g, 1.30 mmol) was dissolved in 4 mL CHCl_3_. A drop of DMF was added to the solution followed by thionyl chloride (400 μL, 5.5 mmol). The reaction mixture was heated to reflux and maintained at that temperature for 1 h. Then, the solvent was removed under reduced pressure to dryness. The crude acid chloride was then dissolved in 4 mL dry DCM, the solution was cooled to 0 °C, and l-threonine methyl ester HCl (0.227 g, 1.30 mmol), Et_3_N (373 µL, 2.60 mmol), and DMAP (16 mg, 0.13 mmol) were added. The mixture was allowed to warm to rt, with stirring, over 3 h. Following, the DCM was removed under reduced pressure, and the residue was dissolved in ethyl acetate (20 mL). The solution was then washed with 1M HCl (1 × 20 mL) and saturated NaHCO_3_ (1 × 10 mL). The organic layer was dried with Na_2_SO_4_, filtered, and the solvent was evaporated under reduced pressure. The residue was purified by column chromatography (10% -30% EtOAc:Hexanes) to deliver 437.9 mg (90%) of compound **10** as a colorless oil. R_f_ 0.16 (40% EtOAC:Hexanes); ^1^H NMR (400 MHz, CDCl_3_) δ 8.90 (d, J = 8.4 Hz, 1H), 7.81 (dd, J = 7.9, 1.7 Hz, 1H), 7.31 (m, 1H), 7.28 (dd, J = 5.0, 4.0 Hz, 2H), 7.21 (dd, J = 7.9,1.7 Hz 1H), 7.14 (m, 1H), 7.00 (m, 1H), 5.65 (d, J = 12.2 Hz, 1H), 5.62 (d, J = 12.2 Hz, 1H), 5.42 (d, J = 13.6 Hz, 1H), 5.35 (d, J = 13.6 Hz, 1H), 4.84 (dd, J = 8.4, 2.5 Hz, 1H), 4.45 (qd, J = 6.4, 2.5 Hz, 1H), 3.80 (s, 3H), 1.29 (d, J = 6.4 Hz, 3H).^13^C NMR (125 MHz, CDCl_3_) δ171.8, 165.4, 150.1, 149.4,136.4,133.7, 130.2, 129.1, 128.6, 127.9, 126.6, 126.0, 124.9, 123.0, 76.3, 75.1, 68.2, 57.8, 52.5, 20.1; HRMS (ESI-TOF) for C_20_H_22_NO_6_ [M+H]^+^ *m*/*z* calcd 372.1442, obsd. 372.1427.

*o-Xylylenyl-protected oxazolinecarboxylic acid methyl ester* (**11**) This reaction was modified from Kim et al. and is briefly described here [30]. In a 100 mL round bottom flask, compound **10** (170.1 mg, 0.45 mmol) and MoO_2_(acac)_2_ (0.0073 g, 0.02 mmol) were dissolved in 100 mL toluene. The flask was fitted with a Soxhlet extractor filled with 4Å molecular sieves. The solution was refluxed for 5 h, then allowed to cool to rt and then the solvent was removed under reduced pressure. The crude product was purified by column chromatography (40% EtOAc:Hexanes) to yield compound **11** as a colorless oil (114.2 mg, 70%). R_f_ 0.29 (40% EtOAC:Hexanes); ^1^H NMR (400 MHz, CDCl_3_) δ 7.43 (dd, J = 7.8, 1.7 Hz, 1H), 7.22(m, 4H), 7.10 (dd, J = 8.0, 1.7 Hz, 1H), 6.95 (t, J = 7.9 Hz, 1H), 5.44 (m, 4H), 4.98 (m, 1H), 4.52 (d, J = 7.1 Hz, 1H), 3.83 (s, 3H), 1.56 (d, J = 6.3 Hz, 3H). ^13^C NMR (100 MHz, CDCl_3_) δ 171.7, 164.3, 151.3, 149.3, 136.1, 135.2, 129.4, 128.6, 128.4, 128.4, 124.9, 124.6, 123.4, 121.9, 78.5, 75.9, 75.2, 74.7, 52.5, 21.0; HRMS (ESI-TOF) for C_20_H_20_NO_5_ [M+H]^+^ *m*/*z* calcd 354.1336, obsd. 354.1315.

Preacinetobactin (1) Oxazoline methyl ester **11** (42.1 mg, 0.11 mmol) was dissolved in Et_2_O (10 mL) and KOTMS (16.8 mg, 0.13 mmol) was added. The mixture was stirred at rt for 6 h, then the solvent was removed under reduced pressure. The crude material was used without purification in the subsequent reaction. The dry potassium carboxylate salt (**3a**) and imidazole TFA salt (**4**) (26.1 mg, 0.079 mmol) were dissolved in dry DMF (1 mL) under N_2_ atmosphere and EDC (18 mg, 0.11 mmol) was added. The reaction was stirred overnight at rt and, after, saturated NaHCO_3_ (10 mL) was added to the mixture. This solution was extracted with EtOAc (3 X 10 mL), the organic fractions were combined, dried with Na_2_SO_4_, filtered, and the solvent was removed under reduced pressure. The residue was purified by column chromatography (3% MeOH:DCM) to afford protected preacinetobactin (20.7 mg, 49%) as a colorless oil. The protected preacinetobactin (20.0 mg, 0.037 mmol) was put in a dry flask under N_2_. Pd/C (1 mg) was added to the flask, followed by the slow addition of dry methanol (1 mL) and TFA (100µL). The flask was purged with H_2_ once and was put under an atmosphere of H_2_ and the reaction was allowed to stir overnight at rt. The reaction mixture was then filtered through a short pad of celite, and the filtrate was concentrated to dryness to yield 12.6 mg (97%) of preacinetobactin **1** as a purple oil. ^1^H NMR (300 MHz, CD_3_OD) δ 8.78 (s, 1H), 7.37 (s, 1H), 7.17 (d, J = 7.7 Hz, 1H), 6.99 (d, J = 7.4 Hz, 1H), 6.76 (t, J = 8.4 Hz, 1H), 5.09 (d, J = 5.9 Hz, 1H), 4.07 (m, 1H), 3.98 (m, 1H), 3.12 (m, 2H), 1.52 (d, J = 6.2 Hz, 3H). ^13^C NMR (100 MHz, CD_3_OD) δ 169.6, 167.6 150.1, 146.0, 133.7, 130.7, 122.1, 120.0, 119.9, 116.5, 106.4, 84.2, 64.9, 21.4, 19.0; HRMS (ESI-TOF) for C_16_H_19_N_4_O_5_ [M+H]^+^ *m*/*z* calcd 347.1350, obsd. 347.1323.

*5-Bromo-o-vanillin* (**13**) This procedure follows the one previously reported in work by Jung et al. [31]. *Ortho*-vanillin **12** (1.00 g, 6.50 mmol) was dissolved in acetic acid (20 mL) and the solution was cooled to 0 °C on an ice bath. To the solution was added sodium acetate (0.592 g, 7.20 mmol) followed by dropwise addition of Br_2_ (372 μL, 7.20 mmol). The reaction was allowed to warm to rt while stirring for 30 min. Water (50 mL) was slowly added to the reaction mixture and then it was extracted with DCM (3 × 15 mL). The combined organic layers were washed with brine (1 × 15 mL) then dried with Na_2_SO_4_, filtered, concentrated to dryness to yield pure **13** (1.48 g, 98%) as a dark brown solid. R*_f_* 0.53 (30% EtOAc:Hexanes); ^1^H NMR (400 MHz, CDCl_3_) δ 11.0 (s, 1H), 9.88 (s, 1H), 7.33 (d, J = 2.2 Hz, 1H), 7.20 (d, J = 2.1 Hz, 1H), 3.94 (s, 3H); ^13^C NMR (100 MHz, CDCl_3_) δ 195.6, 151.1, 149.5, 126.3, 121.5, 121.0, 111.2, 56.8; HRMS (ESI-TOF) for C_7_H_8_BrO_3_ [M+H]^+^ *m/z* calcd 230.9651, obsd. 230.9625.

*5-Bromo-2,3-dihydroxybenzaldehyde* (**14**) This procedure follows the one previously reported by Yamaguchi et al. [32]. Under an N_2_ atmosphere, 5-bromo-*o*-vanillin **13** (930.9 mg, 4.06 mmol) was dissolved in 11 mL dry DCM. The solution was cooled to -78 °C in a dry ice-acetone bath and BBr_3_ (1.50 mL, 16.3 mmol) was slowly added dropwise. The mixture was allowed to warm to rt with stirring over 6 h. After, saturated NaHCO_3_ (100 mL) was slowly added to the reaction mixture, the pH was adjusted to pH 9 with 10% NaOH (aq.). The solution was extracted using DCM (3 × 20 mL). The aqueous layer was acidified with 10% HCl to pH 4, then the solution was extracted with EtOAc (3 × 20 mL). The combined EtOAc layers were dried with Na_2_SO_4_, filtered, and the solvent was removed under reduced pressure to yield 813.2 mg (92%) **14** as a pale-yellow solid. R*_f_* 0.35 (30% EtoAc:Hexanes); ^1^H NMR (400 MHz, CD_3_OD) δ 10.0 (s, 1H), 7.33 (d, J = 2.4 Hz, 1H), 7.18 (d, J = 2.4 Hz,1H). ^13^C NMR (100 MHz, CD_3_OD) δ 193.9, 147.2, 123.5, 122.6, 110.5; HRMS (ESI-TOF) for C_8_H_8_BrO_3_ [M+H]^+^ *m*/*z* calcd 216.9500, obsd. 216.9418.

*5-bromo-(2,3-xylylenyl-di-oxy)benzaldehyde* (**15**) 5-Bromo 2,3-dihydroxy benzaldehyde **14** (813.2 mg, 3.74 mmol) and K_2_CO_3_ (1.54 g, 11.2 mmol) were dissolved in 15 mL of DMF in a 50 mL three-neck round bottom flask. The mixture was heated to 130 °C and α,α’-dibromo-*o*-xylene (1.17 g, 4.46 mmol), dissolved separately in an additional 15 mL DMF, was added dropwise to the reaction mixture over a course of 20 min. The reaction was maintained at 130 °C for 1 h then allowed to cool to rt. The reaction mixture was diluted with 100 mL water and the product was extracted with Et_2_O (3 × 10 mL). The combined organic layers were washed with 10% NaOH (2 × 25 mL) and brine (1 × 30 mL). The reaction yielded 1.03 g (87%) **15** as a white solid. mp 145-146 °C. R_f_ 0.63 (40% EtOAc:Hexanes); ^1^H NMR (400 MHz, CDCl_3_) δ 10.3 (s, 1H), 7.61 (d, J = 2.52 Hz, 1H), 7.42 (d, J = 2.52 Hz, 1H), 7.33 (m, 2H), 7.28 (m, 1H), 7.20 (m, 1H), 5.58 (s, 2H), 5.42 (s, 2H); ^13^C NMR (100 MHz, CDCl_3_) 188.3, 152.3, 150.9, 135.5, 133.8, 131.4, 130.2, 129.6, 129.3, 128.9, 128.3, 125.9, 114.9, 76.5, 75.2; HRMS (ESI-TOF) for C_15_H_12_BrO_3_ [M+H]^+^ *m*/*z* calcd 318.9970, obsd. 318.9984.

*5-phenyl-2,3-xylylenyl-di-oxy-benzaldehyde* (**16**) Compound **15** (0.400 g, 1.26 mmol) was dissolved in 1.25 mL EtOH and 2.5 mL toluene. Aqueous Na_2_CO_3_ (2.5 mL, 1 M) was added as well as phenyl boronic acid (0.229 g, 1.89 mmol). Triphenylphosphine (82 mg, 0.030 mmol) and Pd(OAc)_2_ (28 mg, 0.13 mmol) were then added, and the mixture was heated to 80 °C for 15 h. After, it was allowed to cool to rt and then 20 mL water was added. The solution was extracted with EtOAc (2 × 10 mL), and the combined organic layers were washed with brine (1 × 10 mL), dried with Na_2_SO_4_, filtered, and the solvent was removed under reduced pressure. The residue was purified by column chromatography (20-40% EtOAc:Hexanes) to yield 245.6 mg (61%) of **16** as a clear oil. R_f_ 0.64 (40% EtOAc: Hexanes); ^1^H NMR (400 MHz, CDCl_3_) δ10.50 (s, 1H), 7.78 (d, J = 2.4 Hz, 1H), 7.57 (m, 3H), 7.44 (m, 2H), 7.33 (m, 4H), 7.20 (m, 1H), 5.64 (s, 2H), 5.47 (s, 2H); ^13^C NMR (100 MHz, CDCl_3_) δ 189.7,152.5, 150.3, 139.0, 136.04, 136.01, 134.2, 130.1, 129.1, 128.88, 128.81, 128.7, 128.2, 127.6, 127.0, 126.7, 121.4, 76.4, 75.1; HRMS (ESI-TOF) for C_21_H_17_O_3_ [M+H]^+^ *m*/*z* calcd 317.1178, obsd. 317.1141.

*N-[(2,3-o-xylylenyl-di-oxy)-5-phenyl-benzoyl]-l-threonine methyl ester* (**17**) Aldehyde **16** (245.6 mg, 0.77 mmol) was dissolved in 1 mL of acetone and diluted with 1 mL of water. Sulfamic acid (226.1 mg, 2.33 mmol) was added followed by NaClO_2_ (139.2 mg, 1.54 mmol) in portions. The reaction stirred for 1 h, then the solvents were removed under reduced pressure. The white solid was dissolved in EtOAc (20 mL) and washed with water (2 × 10 mL). The organic layer was dried with Na_2_SO_4_, filtered, and the solvent was removed under reduced pressure to afford 5-phenyl-2,3-(xylylenyl-di-oxy)-benzoic acid in quantitative yield (255.6 mg) as a white solid. A sample of the acid (531.2 mg, 1.67 mmol) was then dissolved in 5 mL chloroform and thionyl chloride (250 μL, 3.57 mmol) and one drop of DMF was added to the solution at rt. The mixture was heated to reflux and maintained at that temp for 1 h; the mixture was allowed to cool to rt and then the solvent was evaporated. The resulting acid chloride was dissolved in 4 mL dry DCM, the solution was cooled to 0 °C and l-threonine methyl ester HCl (338 mg, 2.0 mmol), TEA (460 µL, 3.34 mmol), and DMAP (20 mg, 0.16 mmol) were added. The reaction was warmed to rt with stirring over 3 h. The DCM was evaporated, and the residue was dissolved in 30 mL EtOAc. The solution was then washed with 1M HCl (1 × 20 mL) and saturated NaHCO_3_ (1 × 10 mL). The organic layer was dried with Na_2_SO_4_, filtered, and the solvent was evaporated under reduced pressure. The residue was purified by column chromatography (10% to 30% EtOAc:Hexanes) to deliver 528.6 mg (70%) of compound **17** as a colorless oil. R_f_ 0.09 (40% EtOAc:Hexanes); ^1^H NMR (400 MHz, CDCl_3_) δ 8.93 (d, J = 8.36 Hz, 1H), 8.11 (d, J = 2.44 Hz, 1H), 7.57 (m, 2H), 7.49 (d, J = 2.44 Hz, 1H), 7.41 (m, 2H), 7.32 (m, 5H), 7.17 (m, 1H), 5.70 (d, J = 12.3 Hz, 1H), 5.66 (d, J = 12.3 Hz, 1H), 5.48 (dd, J = 13.68 Hz, 1H), 5.40 (dd, J = 13.68 Hz, 1H), 4.89 (dd, J = 8.4, 2.3 Hz, 1H), 4.47 (qd, J = 6.36, 2.48 Hz, 1H), 3.84 (s, 3H), 1.33 (d, J = 6.4 Hz, 3H); ^13^C NMR (100 MHz, CDCl_3_) δ 165.4, 150.3, 148.6, 139.2, 136.3, 136.0, 133.8, 130.2, 129.2, 128.9, 128.79, 128.73, 127.9, 127.4, 126.7, 125.07, 125.01, 124.3, 75.2, 68.3, 57.8, 52.5, 20.1; HRMS (ESI-TOF) for C_26_H_26_NO_6_ [M+H]^+^ *m*/*z* calcd 448.1762, obsd. 448.1753.

*o-Xylylenyl-5-phenyl oxazolinecarboxylic acid methyl ester* (**18**) In a 250 mL round bottom flask, methyl ester **17** (528.6 mg, 1.18 mmol) and MoO_2_(acac)_2_ (46 mg, 0.143 mmol) were dissolved in 125 mL toluene. The flask was fitted with a Soxhlet extractor filled with 4Å molecular sieves and the solution was refluxed for 5 h. The mixture was then allowed to cool to rt, and the solvent was removed under reduced pressure. The crude product was isolated by column chromatography (40% EtOAc:Hexanes) to yield 363.3 mg (71%) of **18** as a colorless oil. R_f_ 0.29 (40% EtOAc:Hexanes); ^1^H NMR (400 MHz, CDCl_3_) δ 7.69 (d, J = 2.4 Hz, 1H), 7.54 (m, 2H), 7.41 (m, 2H), 7.36 (d, J = 2.4 Hz, 1H), 7.32-7.21 (m, 5H), 5.52-5.47 (m, 4H), 5.01 (m, 1H), 4.55 (d, J = 7.1 Hz, 1H), 3.85 (s, 3H), 1.58 (d, J = 6.3 Hz, 3H). ^13^C NMR (100 MHz, CDCl_3_) δ 171.7, 164.3, 151.4, 148.5, 139.4, 136.6, 136.1, 135.2, 129.5, 128.7, 128.6, 128.5, 128.4, 127.4, 126.8, 123.4, 123.1, 122.1, 78.6, 76.0, 75.2, 74.7, 52.5, 21.0; HRMS(ESI-TOF) for C_24_H_26_NO_5_ [M+H]^+^ *m*/*z* calcd 430.1469, obsd. 430.1503.

*Protected 5-phenyl preacinetobactin* (**20**) Oxazoline methyl ester **18** (45.2 mg, 0.10 mmol) was dissolved in Et_2_O (10 mL) and KOTMS (15.0 mg, 0.11 mmol) was added. The mixture was stirred at rt for 6 h; after, the solvent was removed under reduced pressure and the resulting potassium carboxylate was used without further purification in the subsequent reaction. The carboxylate, DIC (65 µL, 0.42 mmol), and Oxyma (174 mg, 0.31 mmol) were combined and then dissolved in 500 µL dry DMF under an N_2_ atmosphere; this mixture was then stirred for 15 min at rt. In a different flask, the imidazole TFA salt **4** (52.4 mg, 0.16 mmol) was dissolved in dry 500 µL DMF under N_2_ and TEA (43 μL, 0.31 mmol) was added to the solution. The imidazole and TEA solution was then added in one portion to the oxazoline solution, and the mixture was stirred overnight at rt. After, saturated NaHCO_3_ (10 mL) was added to the reaction mixture. This solution was extracted with EtOAc (3 × 10 mL), the organic fractions were combined, dried with Na_2_SO_4_, filtered, and the solvent was removed under reduced pressure. The residue was purified by column chromatography (3% MeOH:DCM) to afford pure **20** (15.2 mg, 25%) as a colorless oil. R_f_ 0.43 (10% MeOH:DCM)^1^H NMR (400 MHz, CDCl_3_) δ 7.67 (d, J = 2.1 Hz, 1H), 7.51 (m, 2H), 7.40-7.24 (m, 13H), 7.09 (m, 1H), 6.82 (s, 1H), 5.47 (m, 4H), 5.1 (m, 2H), 4.92 (m, 1H), 4.84 (m, 1H), 4.01 (s, 2H), 3.04 (m, 2H), 1.36 (d, J = 4.8 Hz, 3H); ^13^C NMR (100 MHz, CDCl_3_) δ 151.4, 139.4, 136.6, 135.1, 129.5, 129.3, 129.0, 128.7, 128.5, 127.4, 126.8, 123.4, 123.1, 75.8, 74.8, 20.4. HRMS (ESI-TOF) for C_37_H_35_N_4_O_5_ [M+H]^+^ *m*/*z* calcd 615.2602, obsd. 615.2606.

*5-Phenyl preacinetobactin* (**21**) Protected pre-acinetobactin **20** (15.2 mg, 0.024 mmol) was put in a dry flask under N_2_ gas. Pd/C (1 mg) was added to the flask, followed by the slow addition of dry methanol (1 mL) and TFA (100µL). The flask was purged with H_2_ gas using balloons, and it was under an atmosphere of H_2_ while the mixture was allowed to stir overnight. The mixture was filtered through a pad of celite; the solvent was removed from the filtrate under reduced pressure to yield 8.0 mg (78%) of 5-phenyl preacinetobactin **21** as a colorless oil. ^1^H NMR (400 MHz, MeOD) δ 8.72 (s, 1H), 7.66 (d, J = 2.8 Hz, 1H), 7.60 (m, 2H), 7.42-7.26 (m, 5H), 5.24 (d, J = 4.2 Hz, 1H), 4.33 (m, 1H), 4.08 (m, 1H), 3.93 (m. 1H), 3.12 (m, 2H), 1.26 (d, J = 8.5 Hz, 3H). ^13^C NMR (100 MHz, MeOD) δ 146.1, 140.2, 133.4, 132.1, 130.8, 128.4, 126.6, 126.1, 116.8, 116.7, 116.5, 116.2, 66.6, 55.2, 21.4, 19.2; HRMS (ESI-TOF) for C_22_H_25_N_4_O_5_ [M+H_3_O]^+^ *m*/*z* calcd 441.1769, obsd. 441.1782 [33].

### 4.2. Metal Binding by Chrome Azural S (CAS) Assay

#### 4.2.1. Preparation of CAS Assay Solution

This procedure was modified from Kim et al. [27]. Hexadecyltirmethylammonium bromide (CTAB) (10.95 mg) was dissolved in warm water and 375 µL of 1 mM FeCl_3_ was added at room temp. To this solution, 1.87 mL of 2 mM aqueous CAS dye was added (Sigma-Aldrich, St. Louis, MO, USA). MES buffer was prepared by adding 2.4g of 2-(*N*-morpholino)ethanesulfonic acid (MES) in 12.5 mL water and 50% wt.% KOH was used to adjust the pH to 5.6. The MES buffer was then combined with the CAS dye solution and the final volume was diluted to 25 mL.

#### 4.2.2. Observation of Metal Binding

To individual wells of a 96-well plate, 50 µL of CAS assay solution was added. To each well was then also added 50 µL of a siderophore solution prepared by diluting a 4 mM siderophore (DMSO) with water so that the final concentrations corresponded to those shown in Table S1. The plate was then heated (37 °C) in a shaker for 3 hrs. Color changes was observed visually and quantified by visible spectroscopy (654 nm) giving absorbances obtained with a plate reader.

### 4.3. Growth/Recovery Assays with A. baumanii ATCC 19606-s1

The following procedure is from Song et al. with minor changes and is described in brief [27]. *A. baumannii* (ATCC 19606-s1) was grown on LB agar plates containing 40 μg/mL kanamycin sulfate, incubated at 37 °C for 24h. From this plate two to three single colonies were used to inoculate a 10 mL liquid culture of LB media supplemented with 40 μg/mL kanamycin sulfate. The liquid culture was incubated at 37 °C with 200 rpm shaking for 24 h. After incubation, the liquid culture was centrifuged down, and the medium was decanted. The bacterial pellet was then resuspended and washed twice with LB media supplemented with 200 μM 2,2′-bipyridine (Bpy), to act as an iron scavenger. The now washed bacterial culture had its optical density measured at 600 nm (OD_600_) and was adjusted to an OD_600_ of 0.1 in 1.5 mL of 200 μM Bpy supplemented media. This now diluted bacterial culture was further diluted to an OD_600_ of 0.005 in 20 mL of Bpy supplemented LB media to be used for inoculation of cultures. Stock solutions of 0.016 M preacinetobactin and derivatives were made in DMSO and were filter sterilized. These solutions were then used to make 1.5 mL of 400 μM preacinetobactin and derivatives in Bpy supplemented LB. These solutions were then serially diluted to give 0.75 mL of two times the final desired drug concentration and were placed in glass culture tubes. The serial dilutions were inoculated with 0.75 mL of diluted bacteria and were incubated at 37 °C with 200 rpm shaking. OD_600_ was monitored to determine extent of growth recovery, and the experiment was repeated in triplicate. The start of exponential growth phase of the bacteria cultures was highly variable, and we were therefore unable to average OD_600_ at specific time points together. In lieu of this the three individual growth curves are presented.

## Data Availability

Data are available from the authors.

## Supplementary Materials

The following supporting information can be downloaded at: https://www.mdpi.com/article/10.3390/molecules27123688/s1. A. Scheme S1: Synthesis of 5-bromo preacinetobactin **S4**. B. Scheme S2: Attempted Suzuki reactions with 5-Bromo preacinetobactin **S4** and 5-bromo oxazoline **S3**. C. Table S1. CAS assay absorbance values. D. Figure S1. Chrome Azurol S iron binding assay results (A 8-hydroxyl quinoline, **21** 5-phenyl preacinetobactin, **1** preacinetobactin). E. Figure S2. UV-visible spectra of 800 μM 5-phenyl preacinetobactin in DMSO. F. Figure S3. UV-visible spectra of 800 μM preacinetobactin derivative in nano pure water. G. Figure S4. Results of growth recovery assay of *A. baumannii* (ATCC 19606-s1, Δ*bas*). H. Synthesis and characterization of intermediates in the synthesis of 5-bromo preacinetobactin **S4**. I. Characterization data for supplementary intermediates in the synthesis of preacinetobactin **1**. J. Characterization data for intermediate **S7** of the 5-phenyl preacinetobactin **(21**) synthesis. K. Figure S5. ^1^H and ^13^C NMR spectra of compound **S2**. L. Figure S6. ^1^H and ^13^C NMR spectra of compound **S3**. M. Figure S7. ^1^H and ^13^C NMR spectra of compound **S4**. N. Figure S8. ^1^H and ^13^C NMR spectra of compound **S5**. O. Figure S9. ^1^H and ^13^C NMR spectra of compound **5**. P. Figure S10. ^1^H and ^13^C NMR spectra of compound **10**. Q. Figure S11. ^1^H and ^13^C NMR spectra of compound **11**. R. Figure S12. ^1^H and ^13^C NMR spectra of compound **S6**. S. Figure S13. ^1^H and ^13^C NMR spectra of compound **1**. T. Figure S14. ^1^H and ^13^C NMR spectra of compound **13**. U. Figure S15. ^1^H and ^13^C NMR spectra of compound **14**. V. Figure S16. ^1^H and ^13^C NMR spectra of compound **15**. W. Figure S17. ^1^H and ^13^C NMR spectra of compound **16**. X. Figure S18. ^1^H and ^13^C NMR spectra of compound **S7**. Y. Figure S19. ^1^H and ^13^C NMR spectra of compound **17**. Z. Figure S20. ^1^H and ^13^C NMR spectra of compound **18**. AA. Figure S21. ^1^H and ^13^C NMR spectra of compound **20**. BB. Figure S22. ^1^H and ^13^C NMR spectra of compound **21**.
