# Supplementary material for: Synthesis and Characterization of Preacinetobactin and 5-Phenyl Preacinetobactin
Source: Molecules. 2022 Jun 9;27(12):3688. doi: 10.3390/molecules27123688 (PMC9227331; doi:10.3390/molecules27123688)

Supporting Information for

Synthesis and characterization of preacinetobactin and 5-phenyl preacinetobactin

Jean M. Bray, Scott Pierce, Alfredo Angeles-Boza, & Mark W. Peczuh*
Dept. of Chemistry, University of Connecticut, 55 N. Eagleville Road, U3060, Storrs, CT 06269 USA

Total Pages: 28

Item	Page(s)
**Scheme S1:** Synthesis of 5-bromo preacinetobactin **S4**	S2
**Scheme S2:** Attempted Suzuki reactions with 5-bromo preacinetobactin **S4** and 5-bromo oxazoline **S3**	S2
**Table S1.** CAS assay absorbance values	S3
**Figure S1.** Chrome Azurol S iron binding assay results (**A** 8-hydroxyl quinoline, **21** 5-phenyl preacinetobactin, **1** preacinetobactin).	S3
UV-Vis Iron Titrations	S4
**Figure S2.** UV-visible spectra of 800 μM 5-phenyl preacinetobactin in DMSO.	S4
**Figure S3.** UV-visible spectra of 800 μM preacinetobactin derivative in nano pure water.	S5
**Figure S4.** Results of growth recovery assay of A. *baumannii* (ATCC 19606-s1, Δ*bas*).	S6
Synthesis and characterization of intermediates in the synthesis of 5-Bromo preacinetobactin **S4**	S7-S8
Characterization data for supplementary intermediates in the synthesis of preacinetobactin **1**	S9
Characterization data for intermediate S7 of 5-phenyl preacinetobactin (**21**) synthesis	S10
**Figure S5.** ^{1}H and ^{13}C NMR spectra of compound **S2**.	S11
**Figure S6.** ^{1}H and ^{13}C NMR spectra of compound **S3**.	S12
**Figure S7.** ^{1}H and ^{13}C NMR spectra of compound **S4**.	S13
**Figure S8.** ^{1}H and ^{13}C NMR spectra of compound **S5**.	S14
**Figure S9.** ^{1}H and ^{13}C NMR spectra of compound **5**.	S15
**Figure S10.** ^{1}H and ^{13}C NMR spectra of compound **10**.	S16
**Figure S11.** ^{1}H and ^{13}C NMR spectra of compound **11**.	S17
**Figure S12.** ^{1}H and ^{13}C NMR spectra of compound **S6**.	S18
**Figure S13.** ^{1}H and ^{13}C NMR spectra of compound **1**.	S19
**Figure S14.** ^{1}H and ^{13}C NMR spectra of compound **13**.	S20
**Figure S15.** ^{1}H and ^{13}C NMR spectra of compound **14**.	S21
**Figure S16.** ^{1}H and ^{13}C NMR spectra of compound **15**.	S22
**Figure S17.** ^{1}H and ^{13}C NMR spectra of compound **16**.	S23
**Figure S18.** ^{1}H and ^{13}C NMR spectra of compound **S7**.	S24
**Figure S19.** ^{1}H and ^{13}C NMR spectra of compound **17**.	S25
**Figure S20.** ^{1}H and ^{13}C NMR spectra of compound **18**.	S26
**Figure S21.** ^{1}H and ^{13}C NMR spectra of compound **20**.	S27
**Figure S22.** ^{1}H and ^{13}C NMR spectra of compound **21**.	S28

Scheme S1: Synthesis of 5-bromo preacinetobactin **S4**

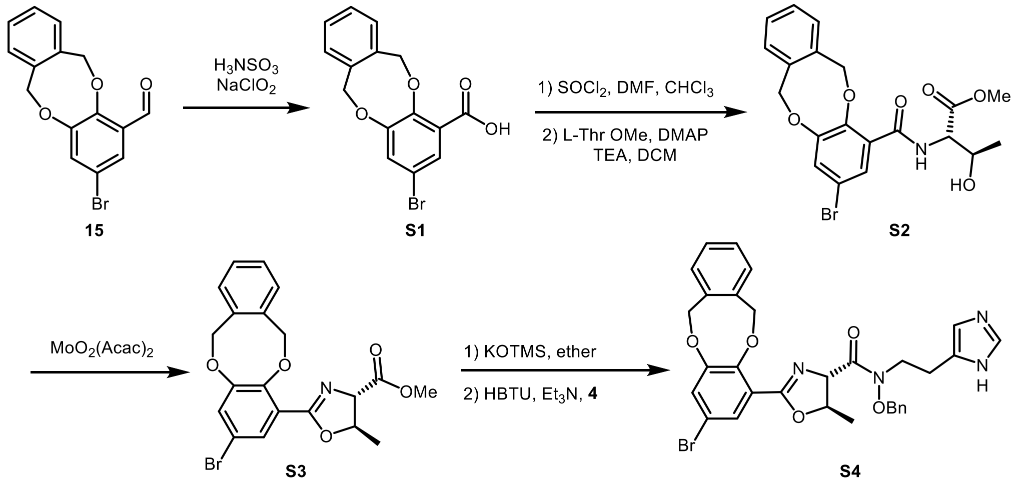

Scheme S2: Attempted Suzuki reactions with 5-bromo preacinetobactin **S4** and 5-bromo oxazoline **S3**

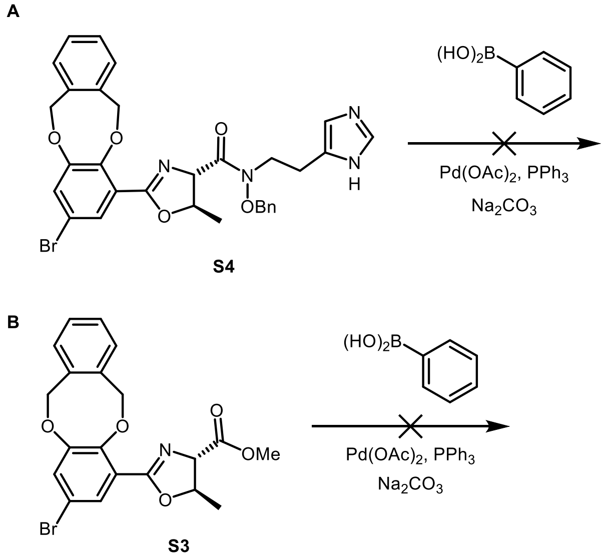

Table S1. CAS assay absorbance values

	Abs. at 654 nm	
[Sider.] μM	5-Phenyl (**21**)	Precinetobacin (**1**)
0	0.187	0.187
10	0.143	0.139
50	0.037	0.039
250	0.036	0.036
1250	0.045	0.038

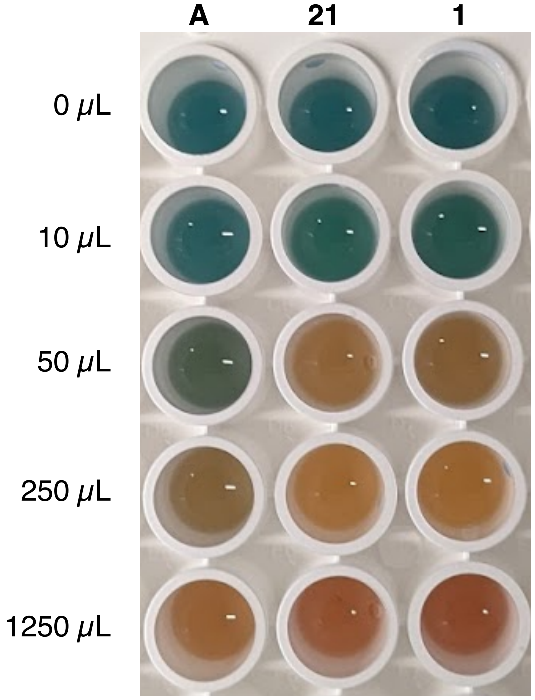

Figure S1. Chrome Azurol S iron binding assay results (**A** 8-hydroxyl quinoline, **21** 5-phenyl preacinetobactin, **1** preacinetobactin).

UV-Vis Iron Titrations

Changes to the UV-vis spectra from 350-800 nm of preacinetobactin **1** and 5-phenyl preacinetobactin **21** were used to assess Fe(III) binding. Measurements were taken on Varian Cary 50 using a quartz cuvette with a pathlength of 1 cm. Stock solutions of **1** and **21** (0.016 M) in DMSO were used to prepare 800 μM working solutions in nano pure water for **1** and pure DMSO for the **21**. DMSO was used for the 5-phenyl derivative due to solubility issues upon Fe(III) binding. A 0.02 M solution of Tris(acetylacetonanto)iron(III) was prepared in methanol to supply Fe(III) for binding. The Fe(III) solution was added in to the preacinetobactin and 5-phenyl derivatives to measure absorbance at 0 μM Fe, 50 μM Fe, 100 μM Fe, 200 μM Fe, 300 μM Fe, 400 μM Fe, and 500 μM Fe. After each addition of the Fe(III) solution the samples were incubated for 5 min to allow for complete Fe(III) binding and the absorbance spectra was measured.

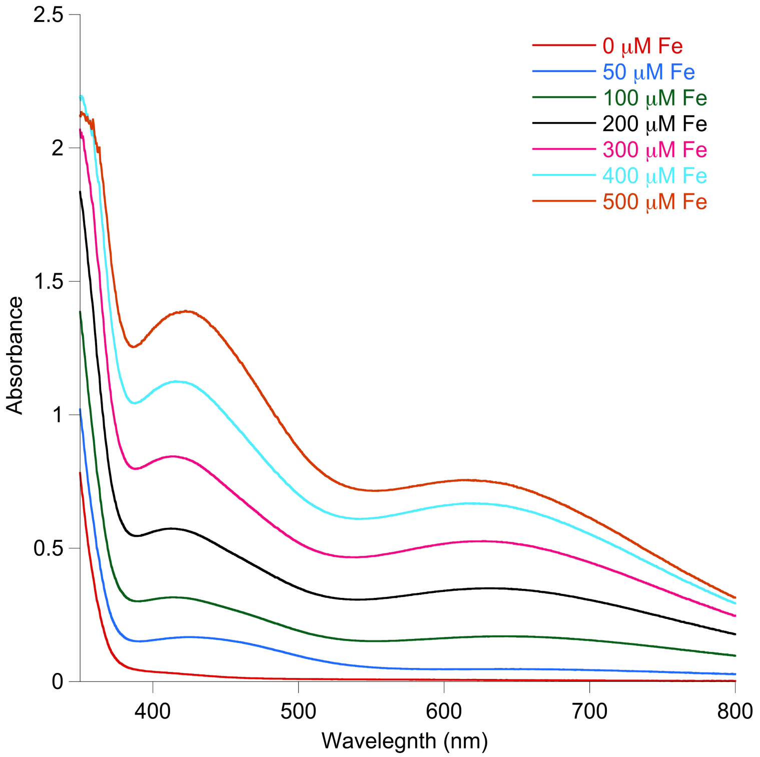

Figure S2. UV-visible spectra of 800 μM 5-phenyl preacinetobactin derivative in DMSO. Fe was supplied through addition of a 0.02 M solution of tris(acetylacetonato)iron(III) in methanol. Solution was allowed to incubate for 5 min after each iron addition before scan was taken.

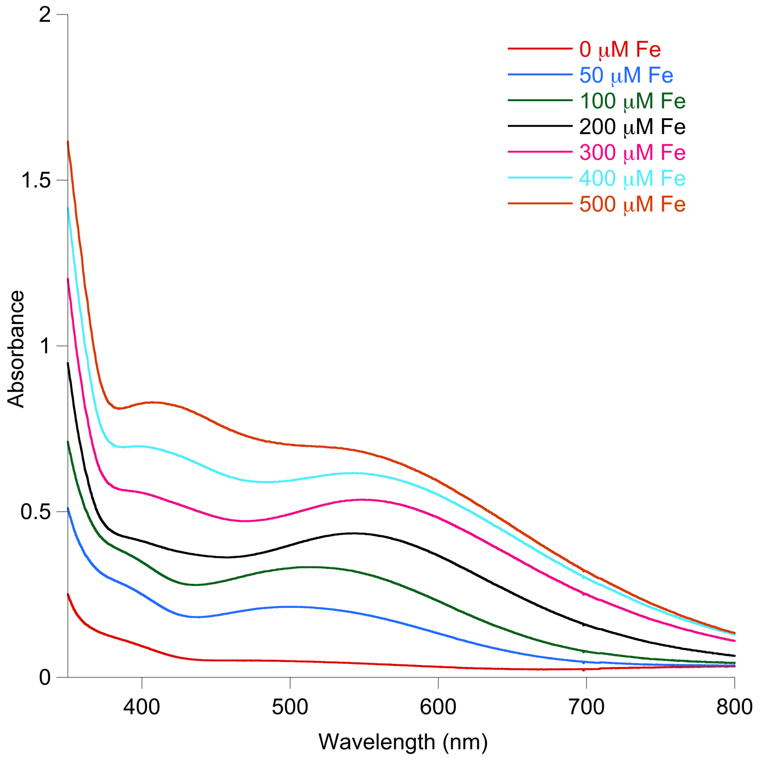

Figure S3. UV-visible spectra of 800 μM preacinetobactin derivative in nano pure water. Fe was supplied through addition of a 0.02 M solution of tris(acetylacetonato)iron(III) in methanol. Solution was allowed to incubate for 5 min after each iron addition before scan was taken.

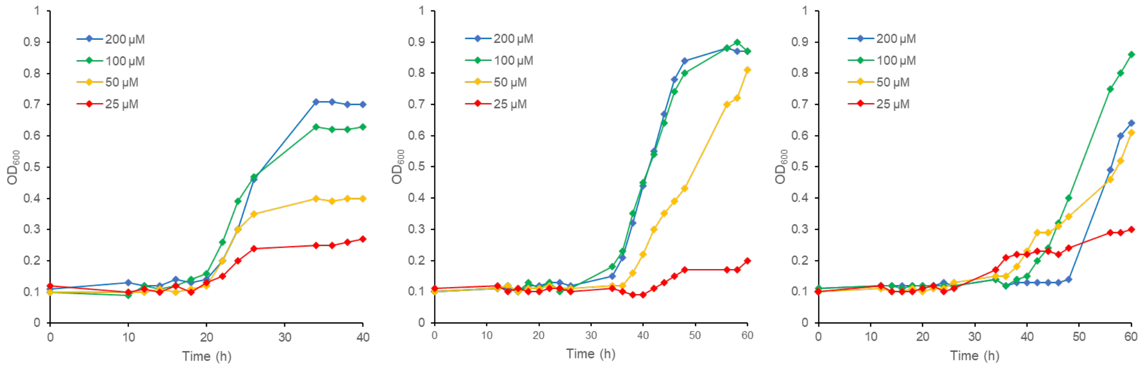

Figure S4. Results of growth recovery assay of A. *baumannii* (ATCC 19606-s1, Δ*bas*) supplemented with preacinetobactin at varying concentrations. Bacteria were grown in LB media supplemented with 200 µM 2,2’-bipyridine. Optical density was measured at 600 nm, with a 1 cm path length.

Synthesis and characterization of intermediates in the synthesis of 5-Bromo preacinetobactin S4

5-Bromo-2,3-(xylylenyl-di-oxy)-benzoic acid (S1)

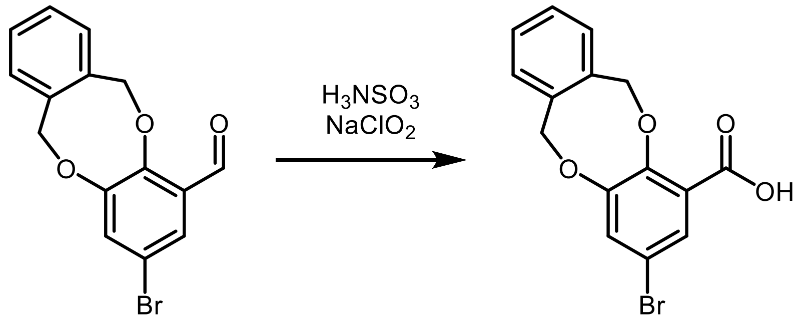

Aldehyde **15** (1.0337 g, 3.26 mmol) was dissolved in 4 mL of acetone and diluted with 4 mL of water. Sulfamic acid (442.1 mg, 4.56 mmol) was added followed by $NaClO_2$ (295.2 mg, 3.26 mmol) in portions. The reaction stirred for 1 h, then the solvents were removed under reduced pressure. The white solid was dissolved in EtOAc (20 mL) and washed with water (2 x 10 mL). The organic layer was dried with Na_2SO_4, filtered, and the solvent was removed under reduced pressure and to afford product **S1** in quantitative yield as a white solid and was moved forward without purification.

N-[(2,3-*o*-xylylenyl-di-oxy)-5-bromo-benzoyl]-L-threonine methyl ester (S2)

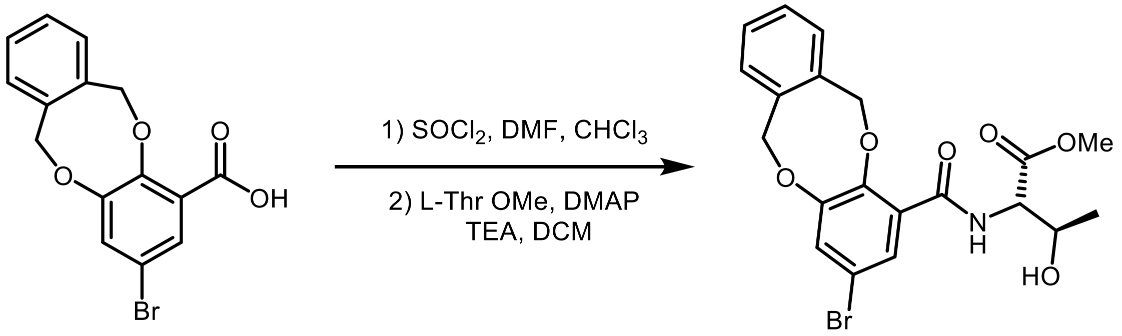

5-Bromo-2,3-(xylylenyl-di-oxy)-benzoic acid **S1** (499.5 mg, 1.5 mmol) was dissolved in 4 mL chloroform. Thionyl chloride (250 µL, 3.57 mmol) and one drop of DMF were added to the solution at rt. The mixture was heated to reflux and maintained at that temp for 1 h; the mixture was allowed to cool back to rt and then the solvent was evaporated. The resulting acid chloride was dissolved in 4 mL dry DCM, the solution was cooled to 0 °C and L-threonine methyl ester HCl (253 mg, 1.5 mmol), TEA (418 μL, 3.00 mmol), and DMAP (18 mg, 0.15 mmol) were added. The reaction was warmed to rt with stirring over 3 h. The DCM was evaporated, and the residue was dissolved in 30 mL EtOAc. The solution was then washed with 1M HCl (1 x 20 mL) and sat'd $NaHCO_3$ (1 x 10 mL). The organic layer was dried with Na_2SO_4, filtered, and the solvent was evaporated under reduced pressure. The residue was purified by column chromatography (30% EtOAc: Hexanes) to deliver 511.6 mg (73%) of compound **S2** as a colorless oil. ^{1}H NMR (400 MHz, $CDCl_3$) δ 8.80 (d, J= 9.1 Hz, 1H), 7.92 (d, J= 2.6 Hz, 1H), 7.31 (m, 4H), 7.15 (m, 1H), 5.64 (d, J= 12.4 Hz, 1H), 5.60 (d, J= 12.4 Hz, 1H), 5.43 (d, J= 13.6 Hz, 1H), 5.34 (d, J= 13.6 Hz, 1H), 4.81 (dd, J= 8.5, 2.4 Hz, 1H), 4.45 (m, 1H), 3.81 (s, 3H), 1.28 (d, J= 6.4 Hz, 3H); ^{13}C NMR (100 MHz, $CDCl_3$) 171.5, 164.1. 150.7, 148.5, 135.9, 133.4, 130.3, 129.4, 129.3, 128.9, 128.1, 126.2, 114.9, 76.4, 75.1, 68.1, 57.8, 52.6, 20.1; HRMS (ESI-TOF) for $C_{20}H_{21}{}^{81}BrNO_6$ $[M+H]^+$ m/z calcd. 452.0529, obsd. 452.0553.

o-Xylylene-protected 5-bromo oxazolinecarboxylic acid methyl ester (S3)

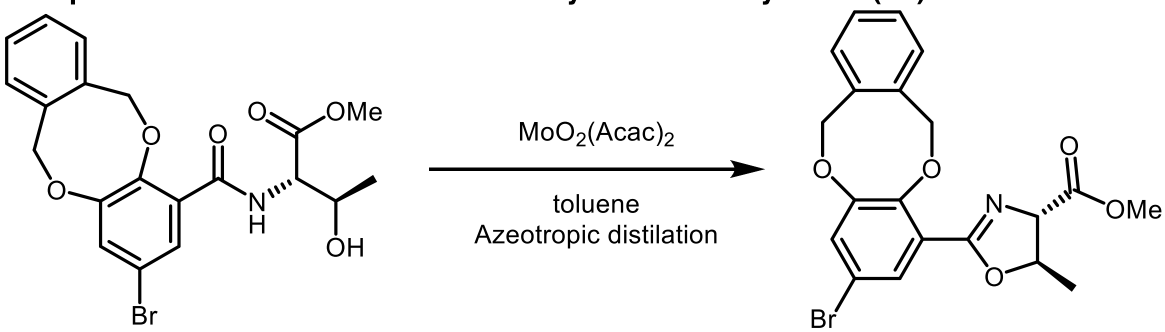

In a 250 mL round bottom flask, methyl ester **S2** (577.5.6 mg, 1.1 mmol) and $MoO_2(acac)_2$ (17 mg, 0.05 mmol)) were dissolved in 125 mL toluene. The flask was fitted with a Soxhlet extractor filled with 4Å molecular sieves and the solution was refluxed for 5 h. The mixture was then allowed to cool to rt, and the solvent was removed under reduced pressure. The crude product was purified by column chromatography (40% EtOAc: Hexanes) to yield 244.9 mg (48%) of **S3** as a colorless oil. ^{1}H NMR (400 MHz, $CDCl_3$) δ 7.56 (d, J= 2.6 Hz, 1H), 7.24 (m, 4H), 7.14 (m, 1H), 5.44 (m, 4H), 4.97 (m 1H), 4.50 (d, J= 6.8 Hz, 1H), 3.82 (s, 3H), 1.53 (d, J= 6.2 Hz, 1H); ^{13}C NMR (100 MHz, $CDCl_3$) δ 171.4, 163.0, 152.0, 148.3, 135.9, 134.5, 129.8, 128.8, 128.6, 128.3, 127.5, 127.4, 123.3, 115.3, 78.7, 75.9, 75.1, 74.5, 52.6, 21.0; HRMS (ESI-TOF) for $C_{20}H_{19}{}^{81}Br\ NO_5$ $[M+H]^+$ *m/z* calcd. 434.0423, obsd.434.0415.

protected 5-bromo preacinetobactin (S4)

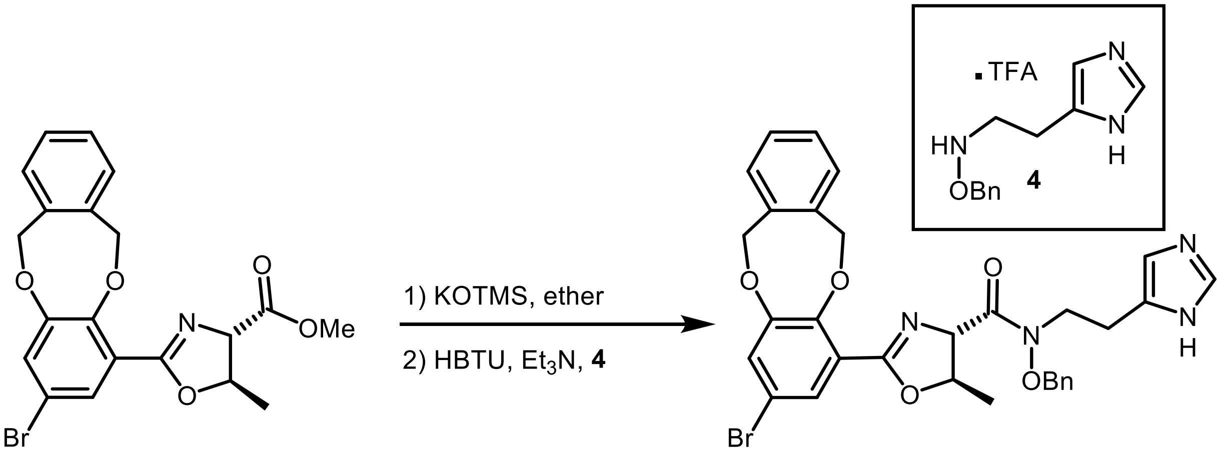

Bromo oxazoline **S3** (179.1 mg, 0.41 mmol) was dissolved in diethyl ether (12 mL) and KOTMS (57.7 mg, 0.45 mmol) was added. The reaction stirred for 5 hrs at room temp until the starting material was consumed. The solution was concentrated and dried under reduced pressure for an hour. The product was then used without purification and was dissolved in 1 mL dry DMF. HBTU (186 mg, 0.49 mmol) was added and the reaction stirred at rt for 5 min. The imidazole TFA salt (108.0 mg, 0.32 mmol) was dissolved in 1 mL dry DMF and added to the oxazoline solution. The reaction stirred overnight at rt. Sodium bicarbonate (10 mL) was added and extracted with ethyl acetate (3 X 10 mL). The combined organic layers were dried on sodium sulfate and concentrated. The crude residue was purified by column chromatography (3% MeOH: DCM) to yield **S4** (115.0 mg, 57%) as a colorless oil. ^{1}H NMR (400 MHz, MeOD) δ 7.78 (s, 1H), 7.48 (m, 2H), 7.43 (m, 4H), 7.27 (m, 4H), 7.18 (m, 1H), 6.96 (s, 1H), 5.46 (m, 4H), 5.05 (s, 2H), 4.68 (m, 1H), 4.23 (m, 1H), 3.89 (m, 1H), 3.02 (m, 2H), 1.34 (d, J= 6.3 Hz, 3H); ^{13}C NMR (100 MHz, MeOD) δ 171.7, 163.3, 151.8, 148.2, 135.6, 134.8, 134.6, 134.3, 133.3, 129.4, 129.3, 128.8, 128.6, 128.6, 128.4, 127.9, 127.5, 126.9, 123.2, 117.0, 114.5, 79.1, 76.2, 74.9, 74.4, 72.0, 60.1, 53.4, 44.9, 39.0, 23.4, 19.4; HRMS (ESI-TOF) for $C_{31}H_{30}{}^{81}Br\ N_4O_5$ $[M+H]^+$ *m/z* calcd. 619.1379, obsd. 619.1328.

Characterization data for supplementary intermediates in the synthesis of preacinetobactin 1

2,3-(*o*-xylylenyl-di-oxy)benzaldehyde (S5)

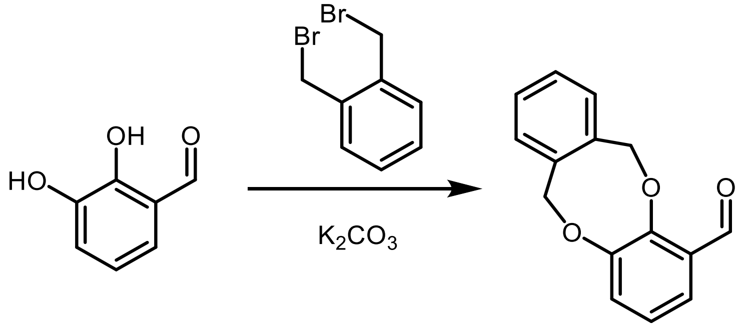

mp 110-112 °C; R_f 0.52 (30% EtOAc:Hexanes); ^{1}H NMR (400 MHz, $CDCl_3$) δ 10.45 (s, 1H), 7.49 (dd, J = 1.64, 7.76 Hz, 1H), 7.27 (m, 4H), 7.14 (m, 1H), 6.95 (t, J = 7.82 Hz, 1H), 5.55 (s, 2H), 5.38 (s, 2H). ^{13}C NMR (100 MHz, $CDCl_3$) δ189.6, 153.2, 150.2, 136.1, 134.2, 130.0, 129.0, 128.8, 128.7, 128.6, 128.1, 123.1, 122.7, 76.3, 75.1; HRMS (ESI-TOF) for $C_{15}H_{13}O_3$ $[M+H]^+$ *m/z* calcd. 241.0859, obsd. 241.0884.

Protected preactinetobactin (S6)

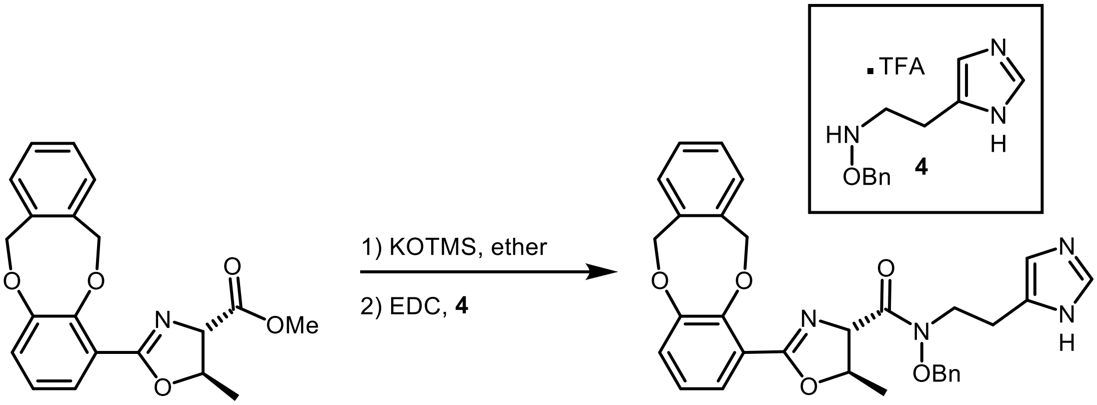

R_f 0.35 (5% MeOH:DCM); ^{1}H NMR (400 MHz, MeOD) δ 7.67 (s, 1H), 7.49 (m, 2H), 7.41 (m, 3H), 7.28 (m, 5H), 7.18 (m, 1H), 7.10 (m, 1H), 6.95 (m, 2H), 5.44 (m, 4H), 5.04 (s, 2H), 4.86 (m, 1H), 4.67 (m, 1H), 4.23 (m, 1H), 3.88 (m, 1H), 3.01 (m, 2H), 1.33 (d, J = 6.2 Hz, 3H); ^{13}C NMR (100 MHz, MeOD) δ 151.2, 149.3, 136.0, 135.6, 129.6, 129.2, 129.0, 128.8, 128.6, 125.0, 124.8, 123.2, 121.8, 79.1, 76.5, 75.0, 74.6, 72.1, 48.7, 19.7; HRMS (ESI-TOF) for $C_{31}H_{31}N_4O_5$ $[M+H]^+$ *m/z* calcd 539.2289, obsd. 539.2253.

Characterization data for intermediate S7 of 5-phenyl preacinetobactin (21) synthesis

5-phenyl-2,3-(xylylenyl-di-oxy)benzoic acid (S7)

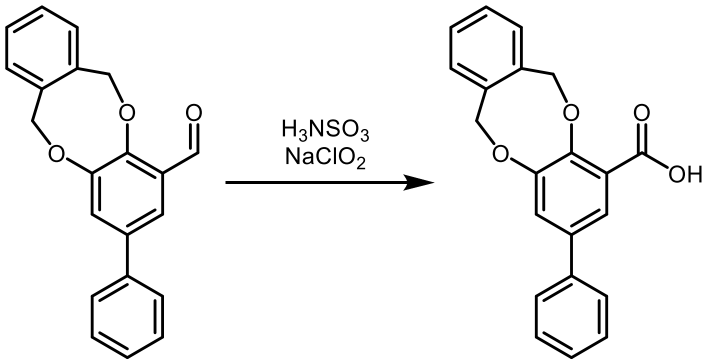

^{1}H NMR (400 MHz, $CDCl_3$) δ 8.13 (d, J = 2.36 Hz, 1H), 7.57 (m, 3H), 7.45 (m, 2H), 7.36 (m, 4H), 7.19 (m, 1H), 5.76 (s, 2H), 5.46 (s, 2H). ^{13}C NMR (100 MHz, $CDCl_3$) δ165.0, 149.6, 148.9, 138.6, 136.8, 136.4, 132.3, 130.8, 129.8, 129.0, 128.9, 127.86, 127.82, 126.7, 126.6, 126.4, 121.2, 75.7. HRMS (ESI-TOF) for $C_{21}H_{17}O_4$ $[M+H]^+$ *m/z* calcd 333.1127, obsd. 333.1133.

Figure S5. ^{1}H & ^{13}C spectra of N-[(2,3-o-xylylenyl-di-oxy)-5-bromo-benzoyl]-L-threonine methyl ester **S2**

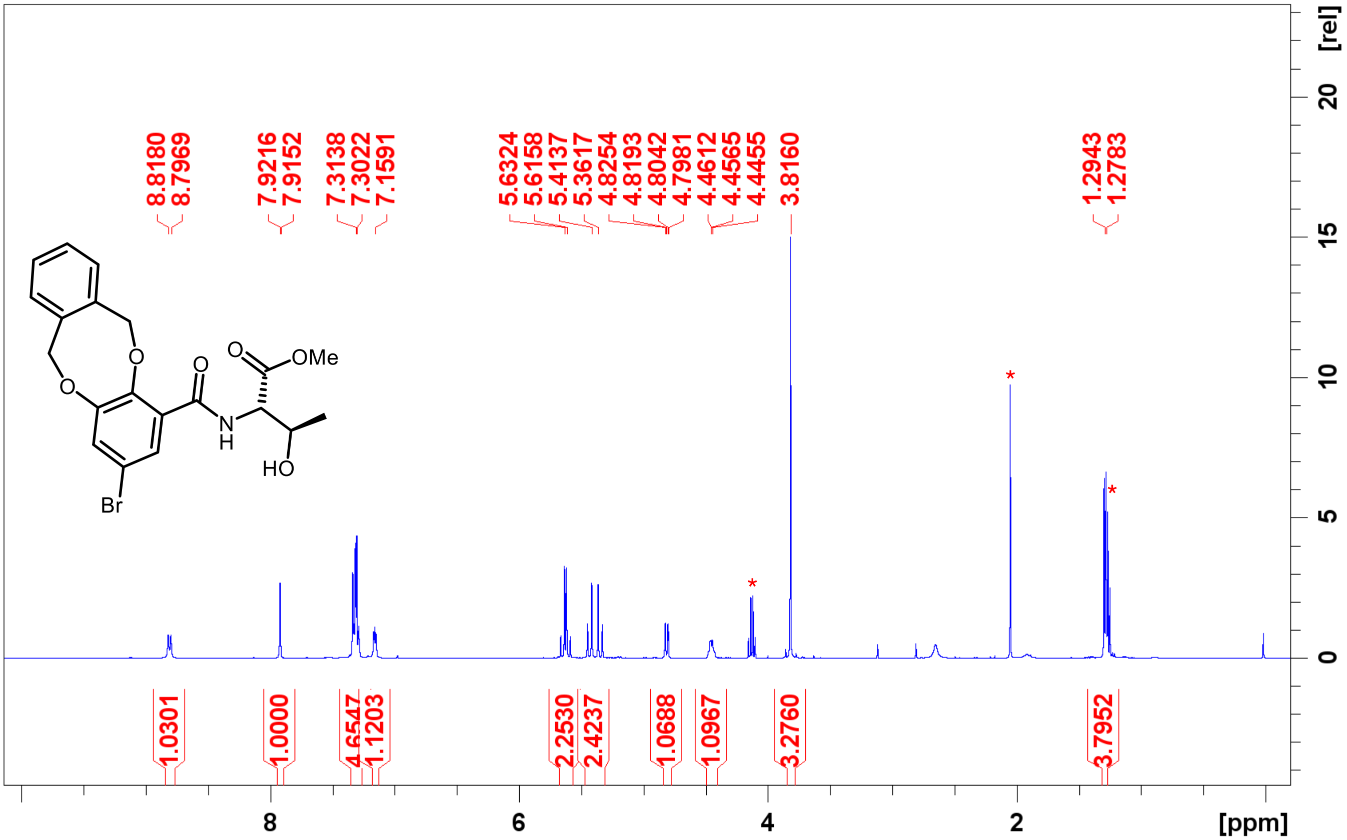

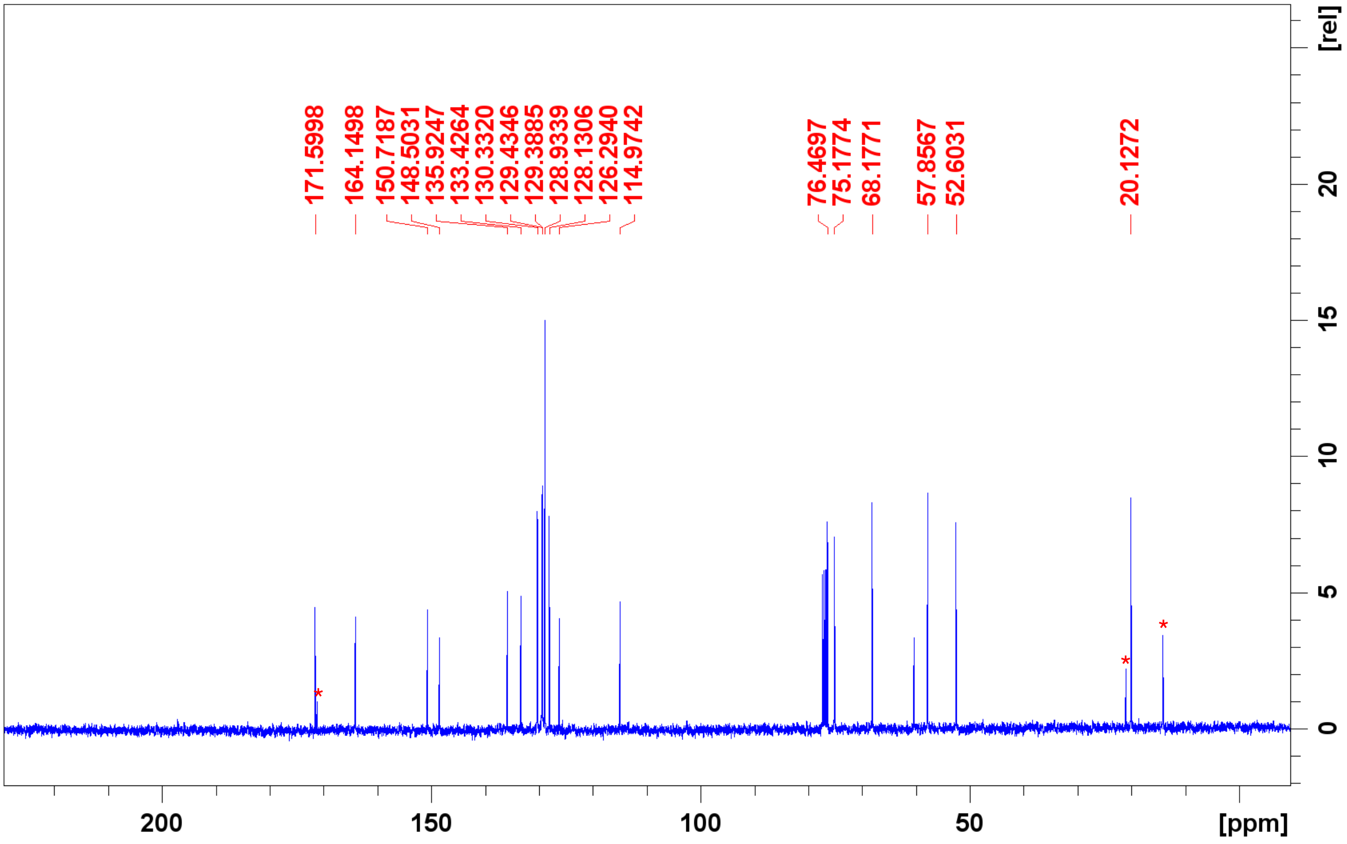

*Ethyl acetate

Figure S6. ^{1}H & ^{13}C spectra of o-Xylylene-protected 5-bromo oxazolinecarboxylic acid methyl ester **S3**

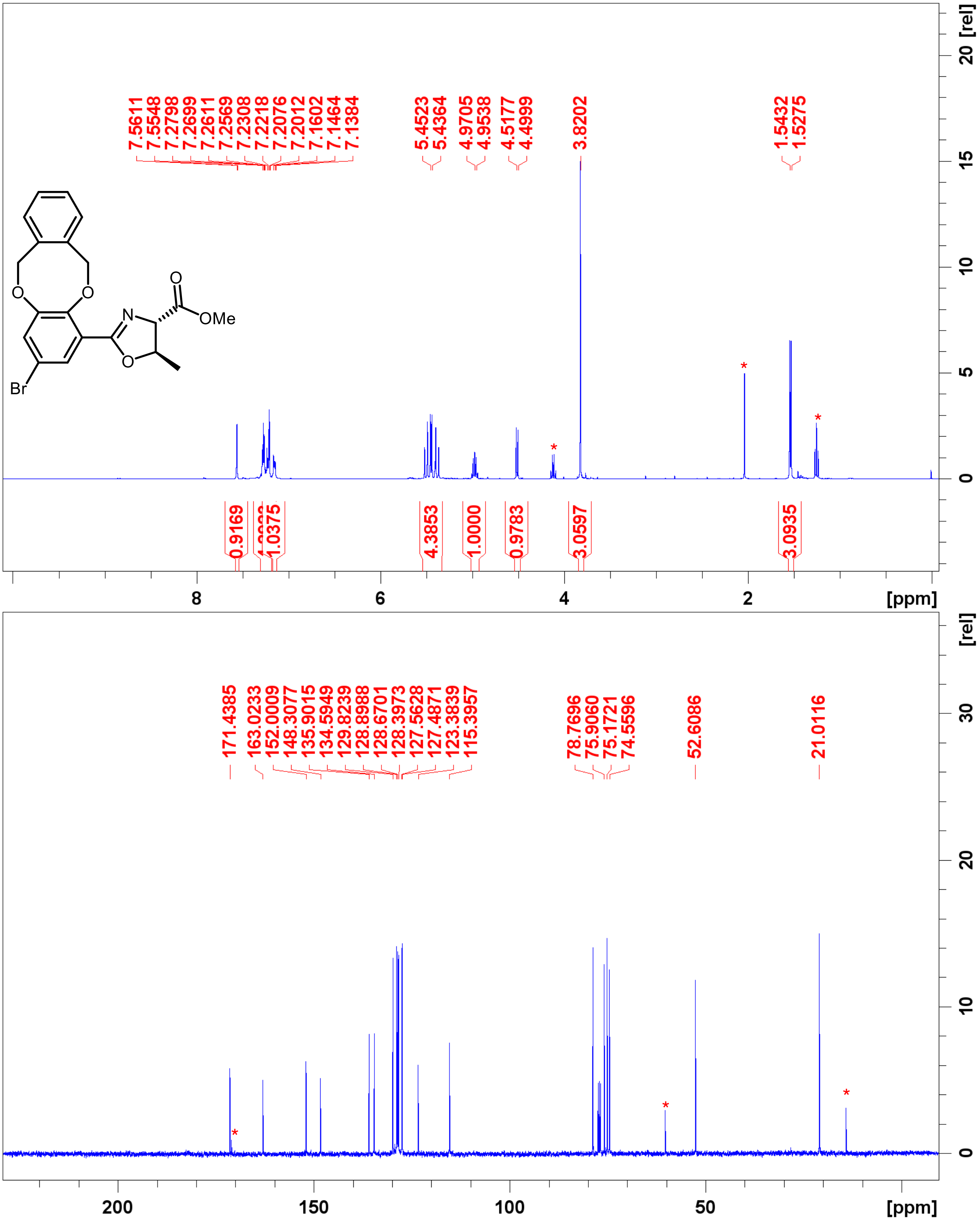

*Ethyl acetate

Figure S7. ^{1}H & ^{13}C spectra of protected 5-bromo preacinetobactin **S4**

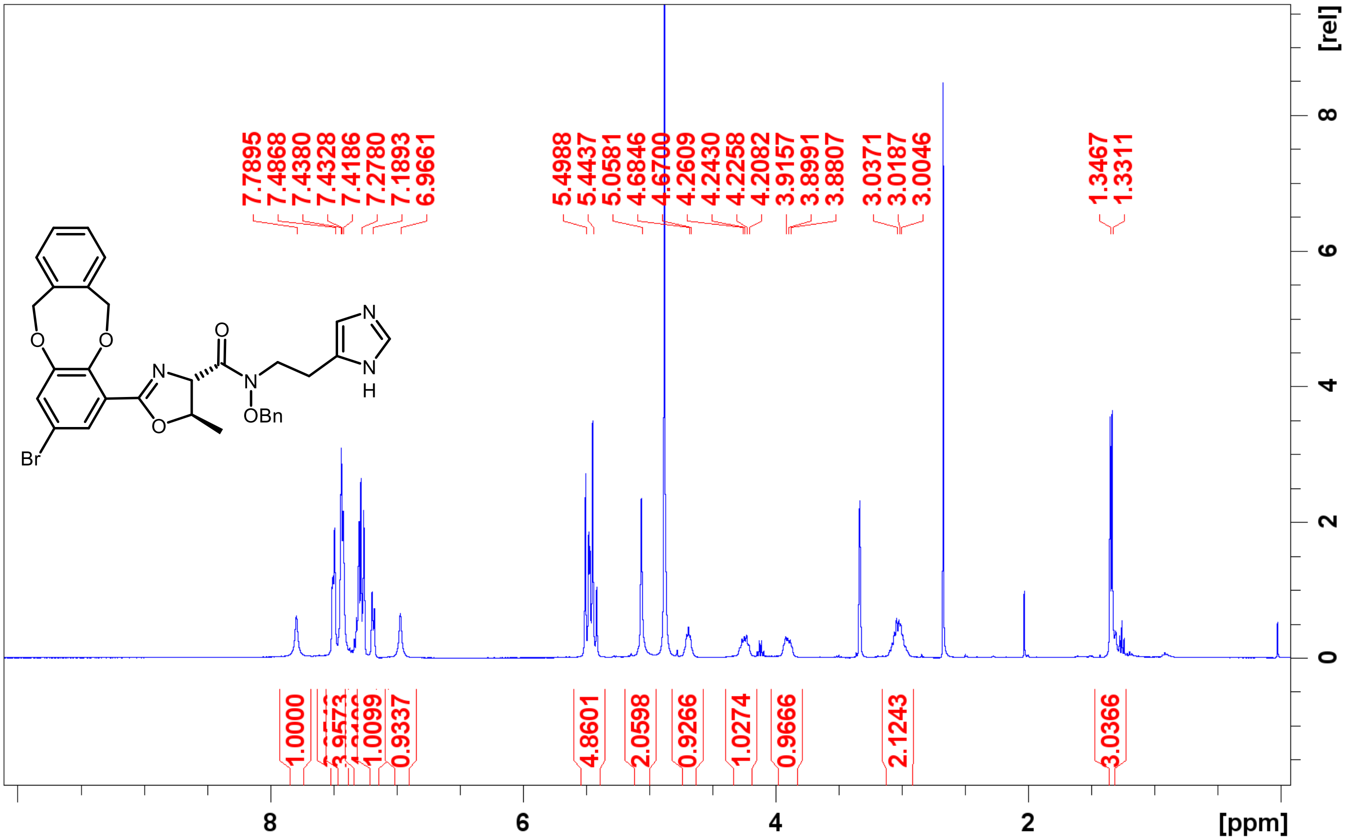

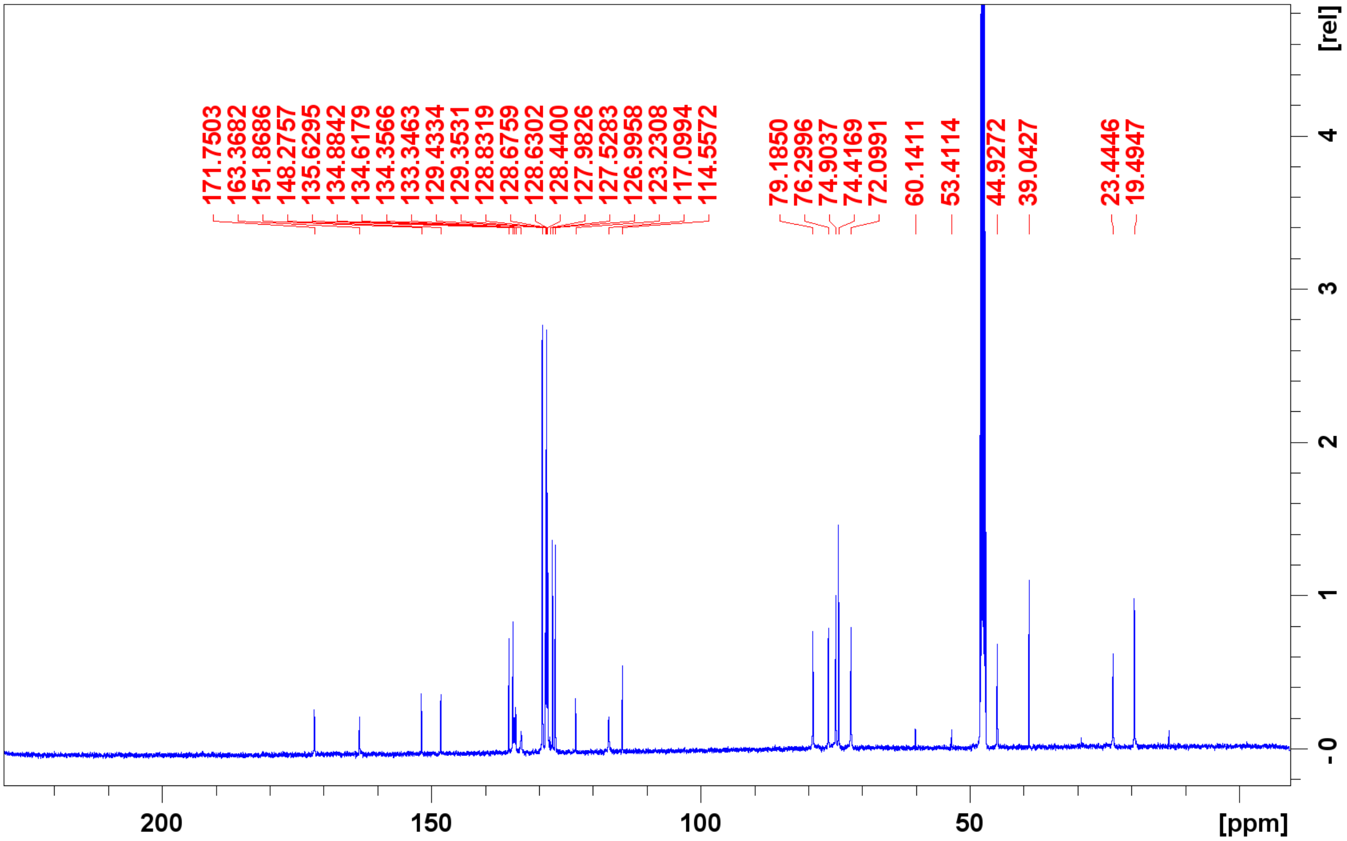

Figure S8. ^{1}H & ^{13}C spectra of compound **S5**.

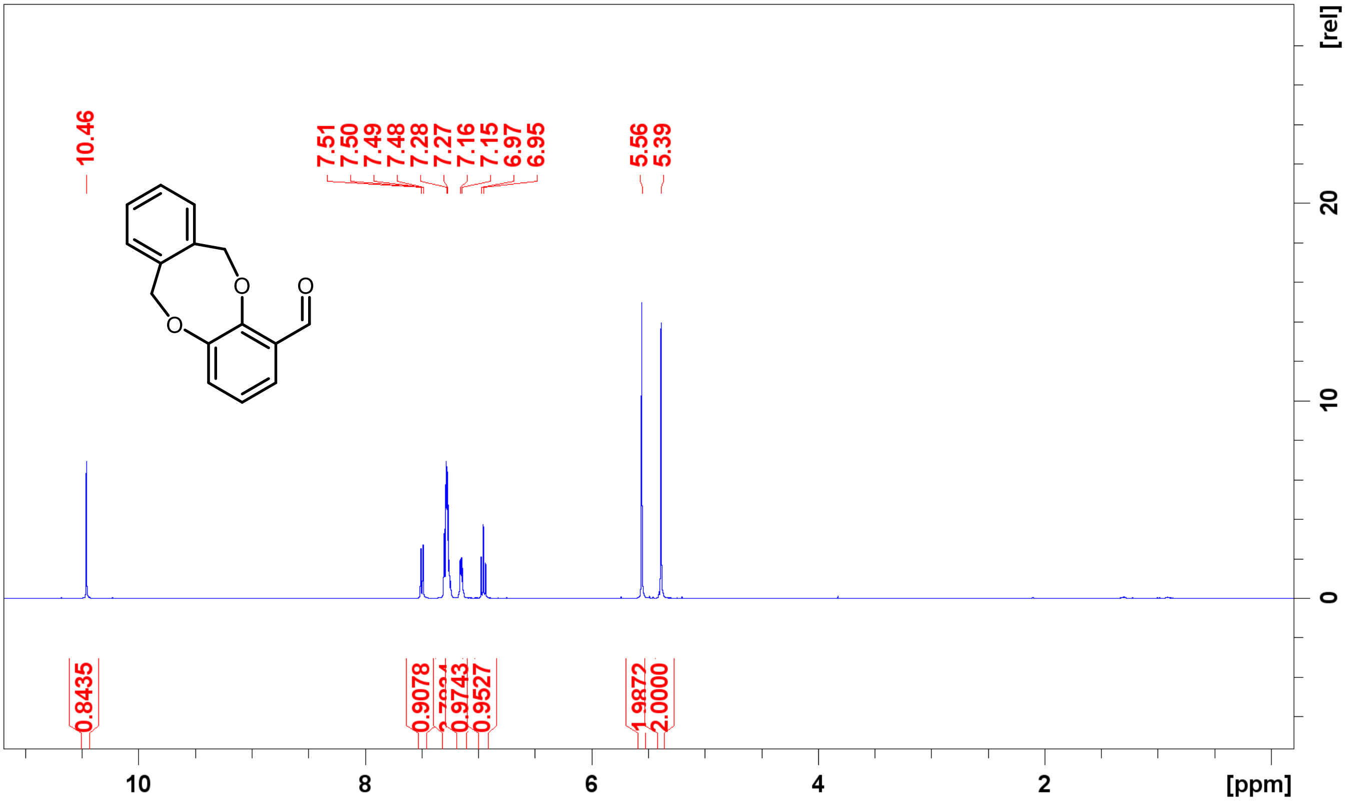

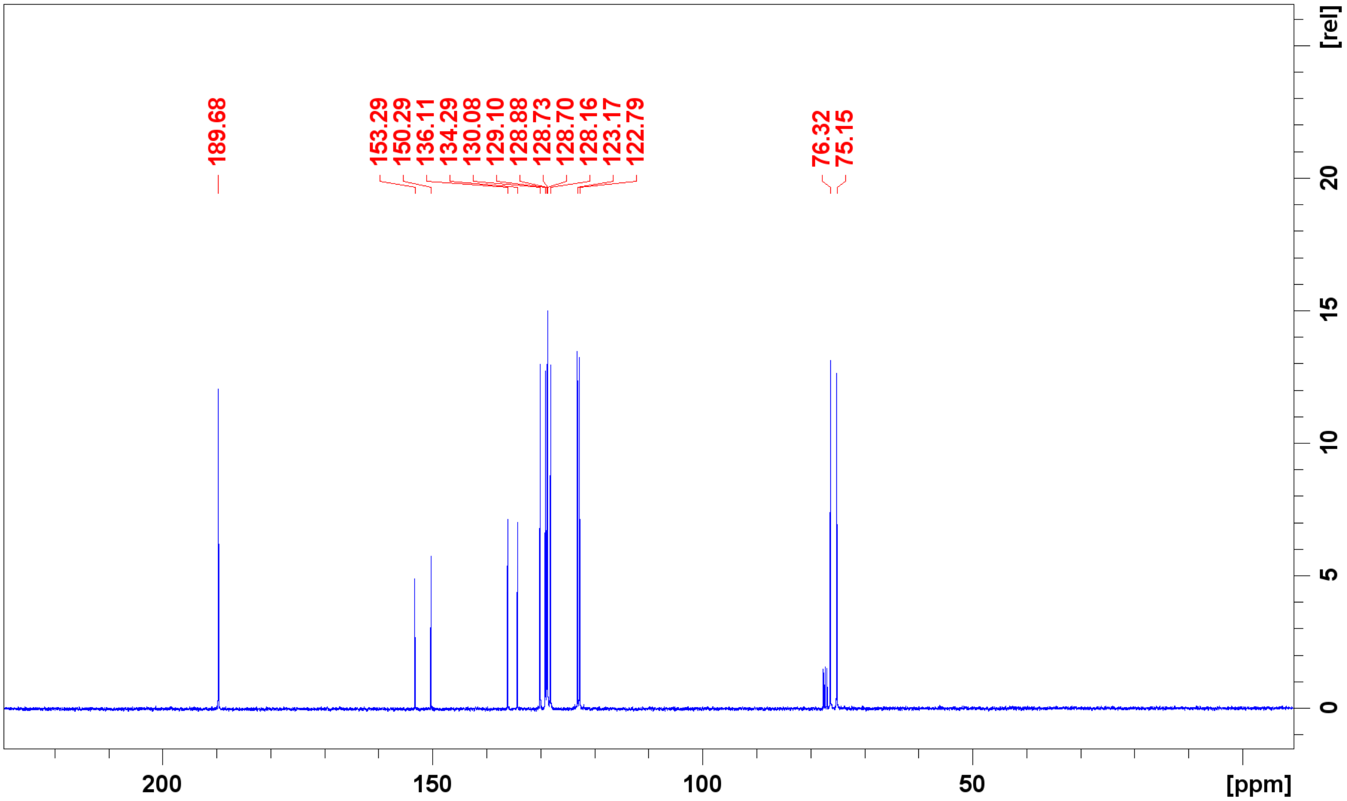

Figure S9. ^{1}H & ^{13}C spectra of compound **5**.

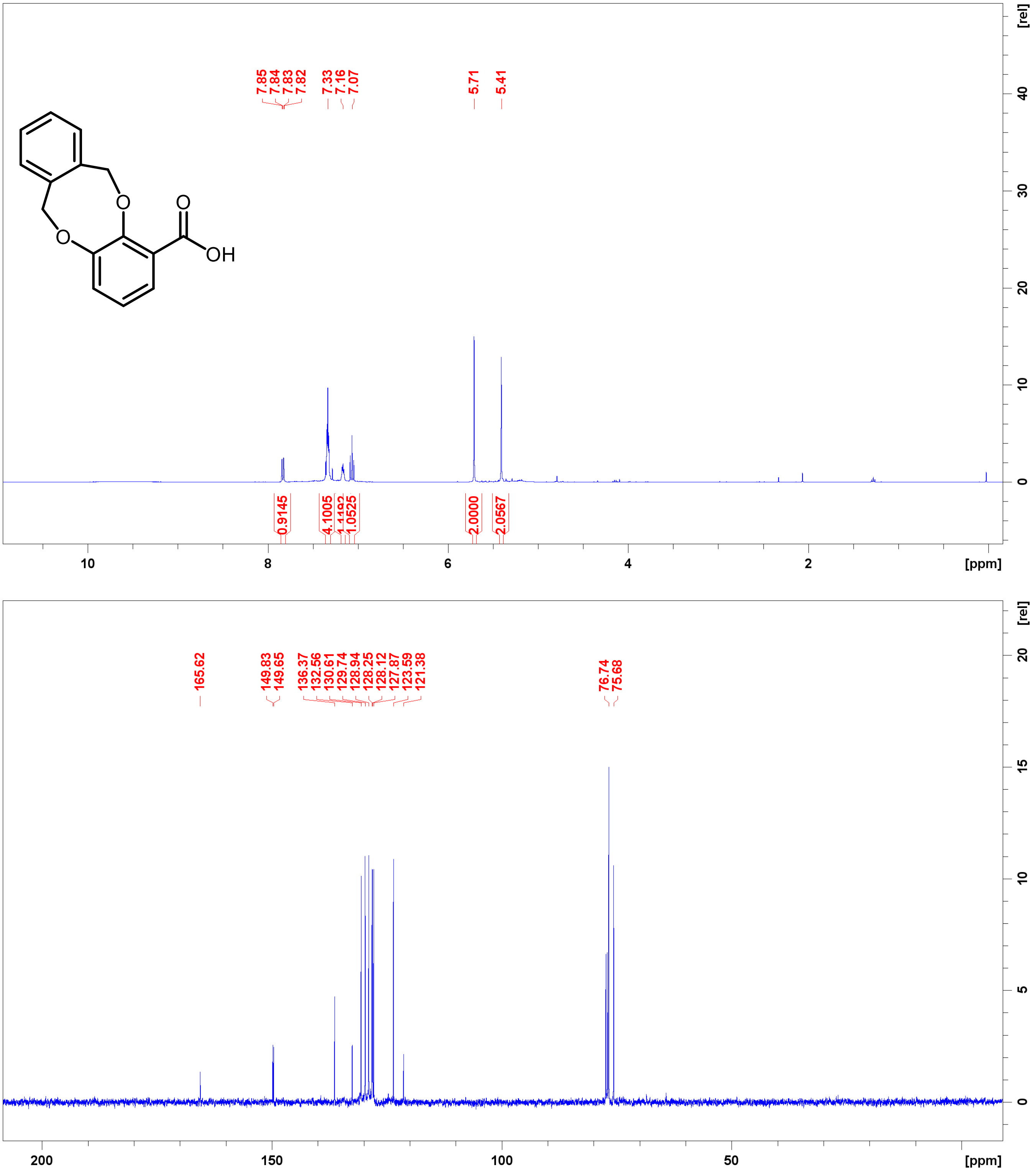

Figure S10. ^{1}H & ^{13}C spectra of compound **10**.

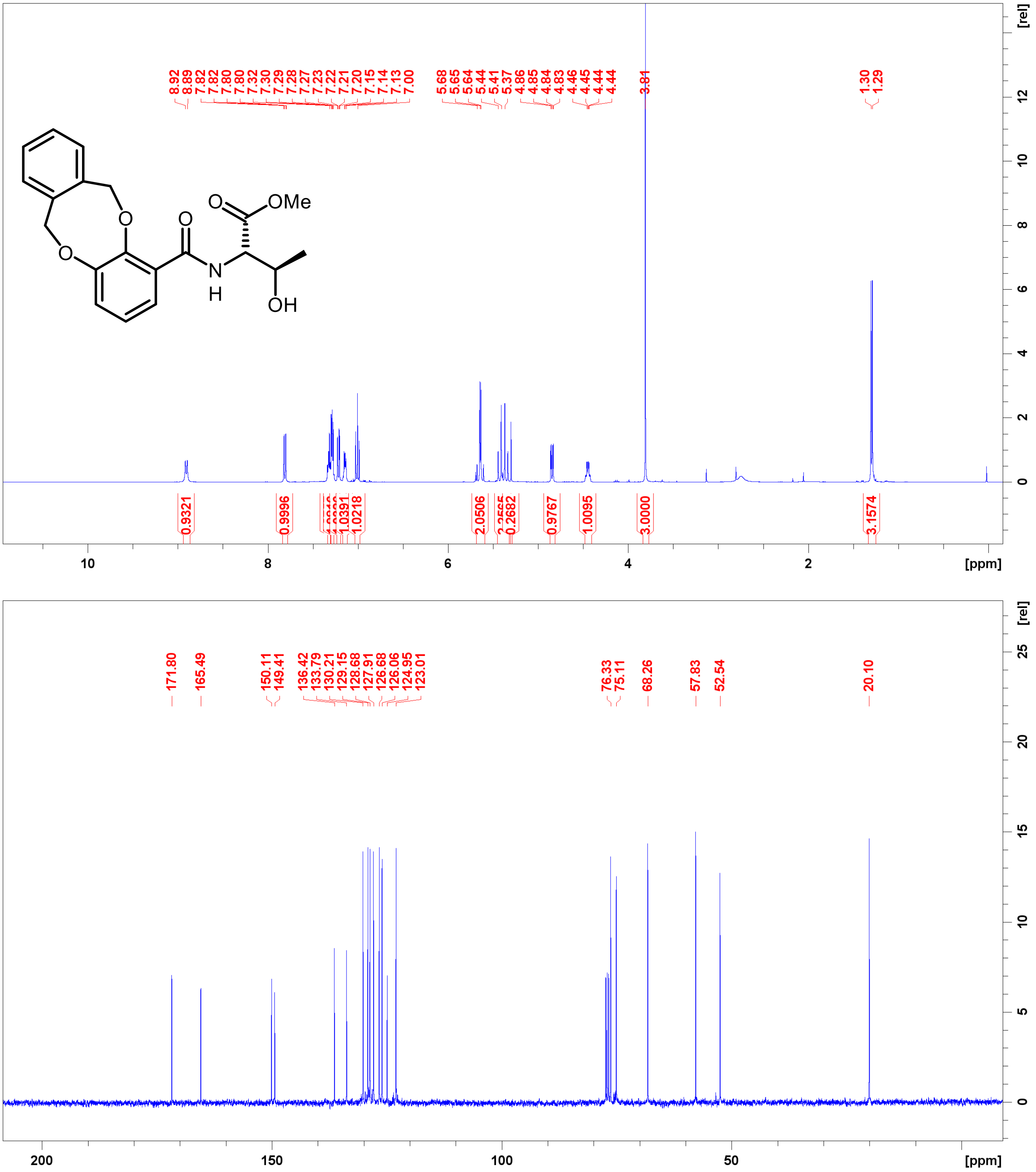

Figure S11. ^{1}H & ^{13}C spectra of compound **11**.

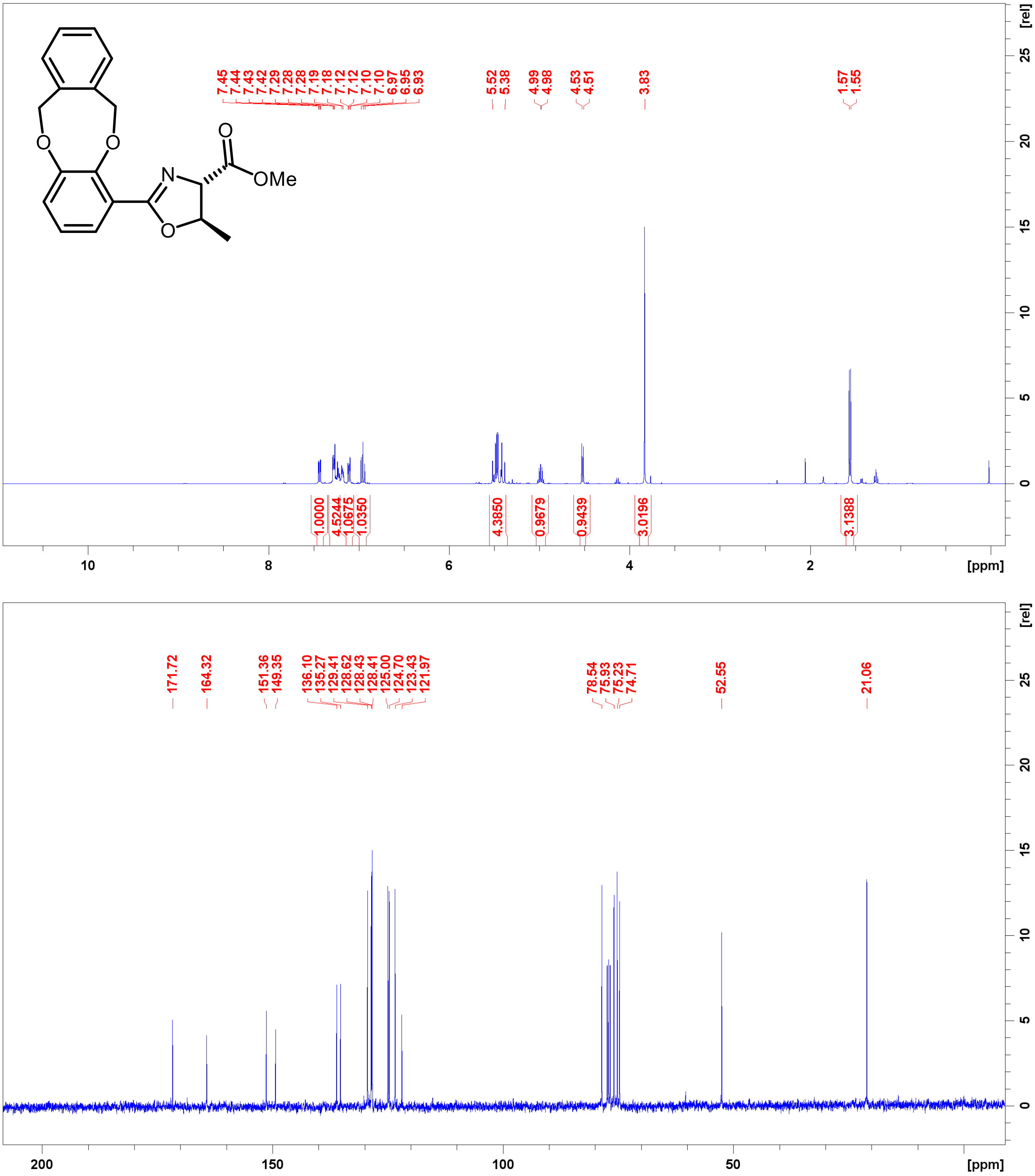

Figure S12. ^{1}H & ^{13}C spectra of compound **S6**.

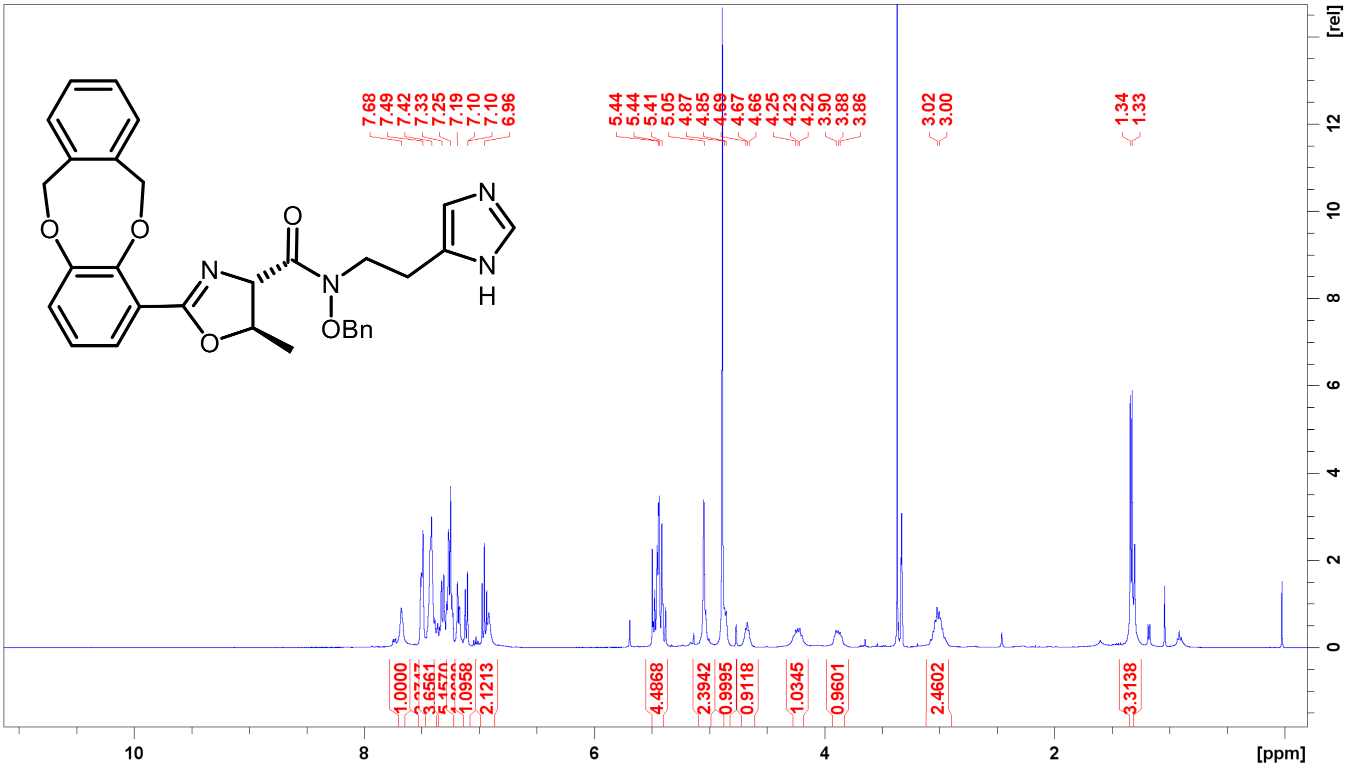

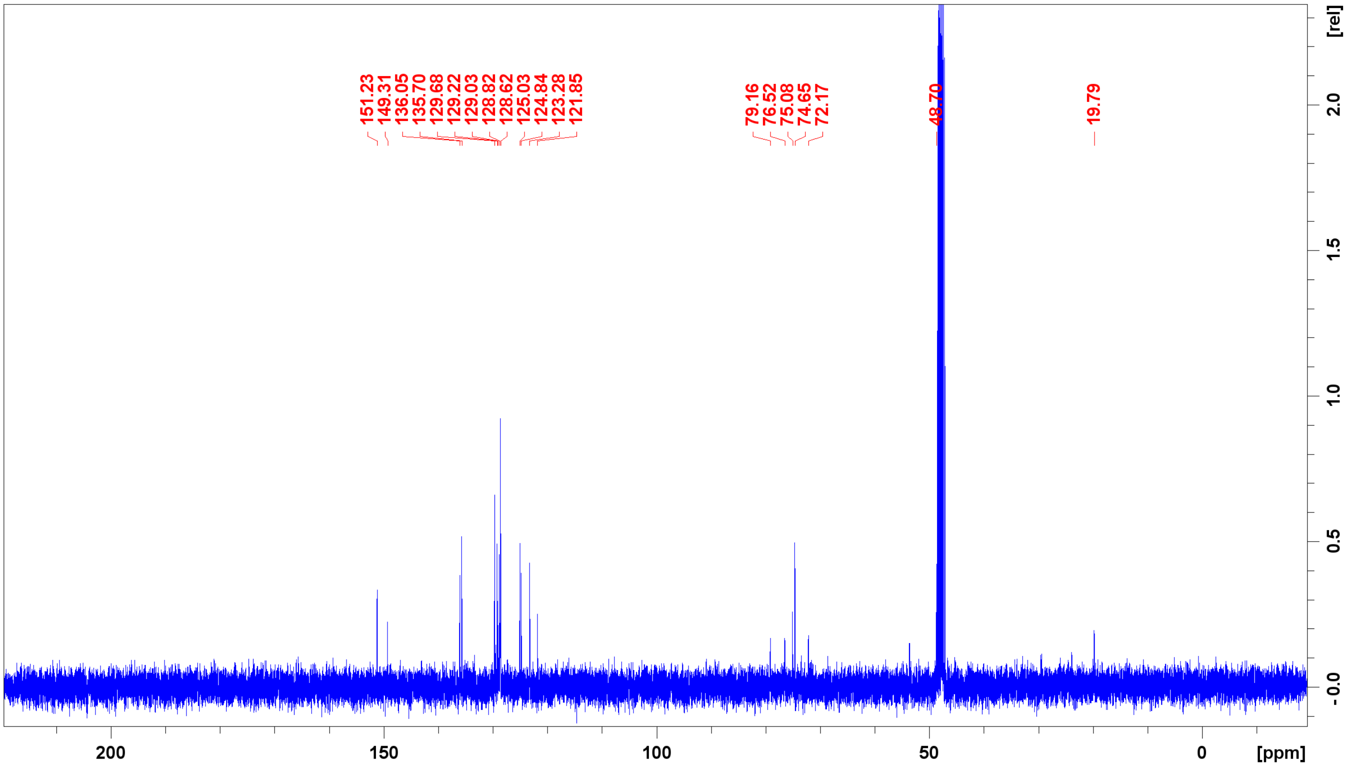

Figure S13. ^{1}H & ^{13}C spectra of compound **1**.

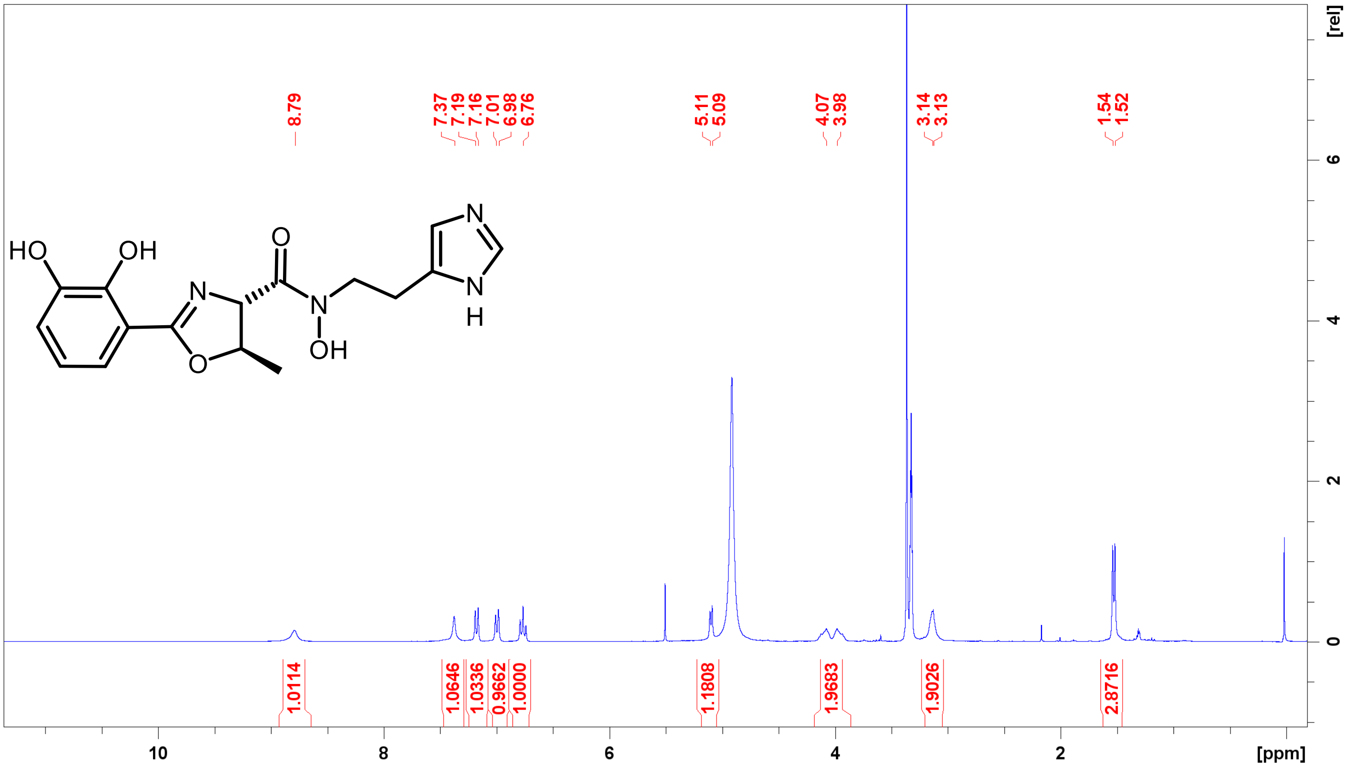

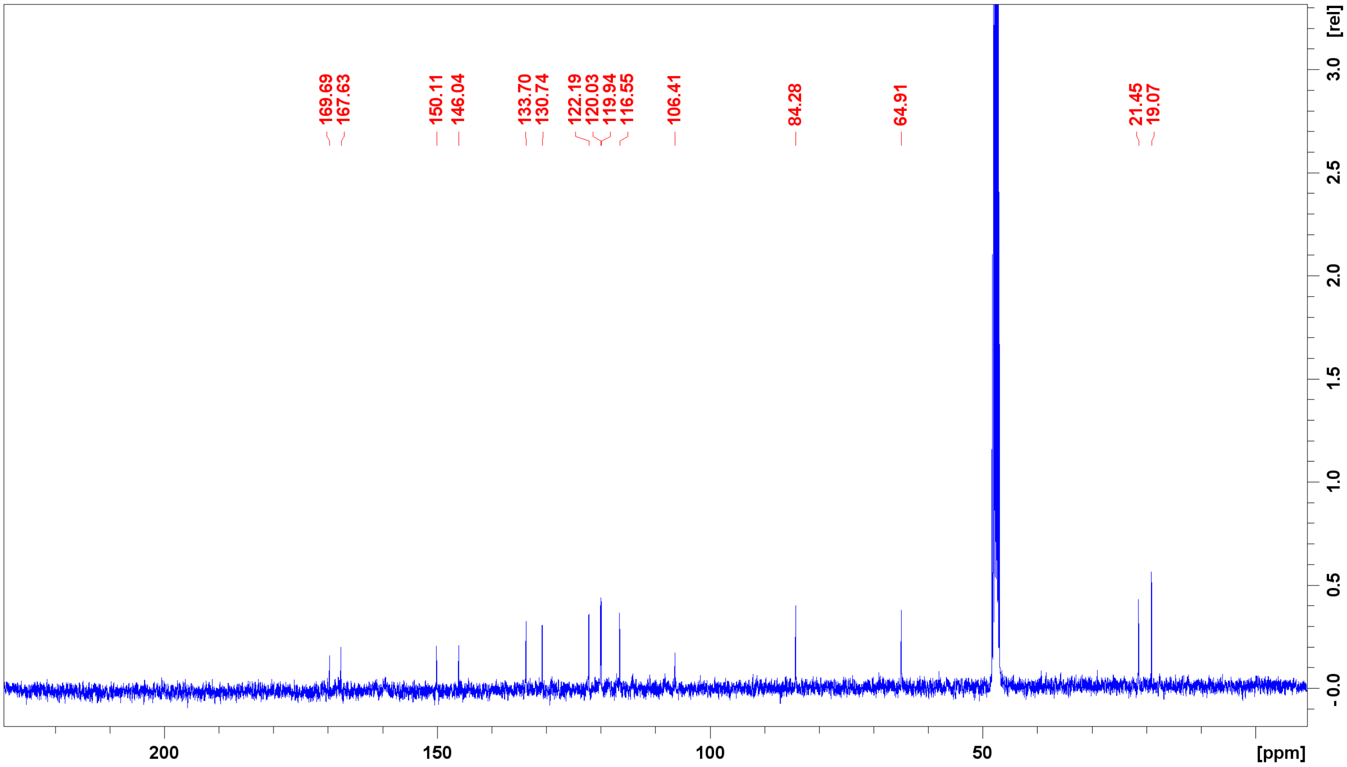

Figure S14. ^1H & ^{13}C spectra of compound **13**.

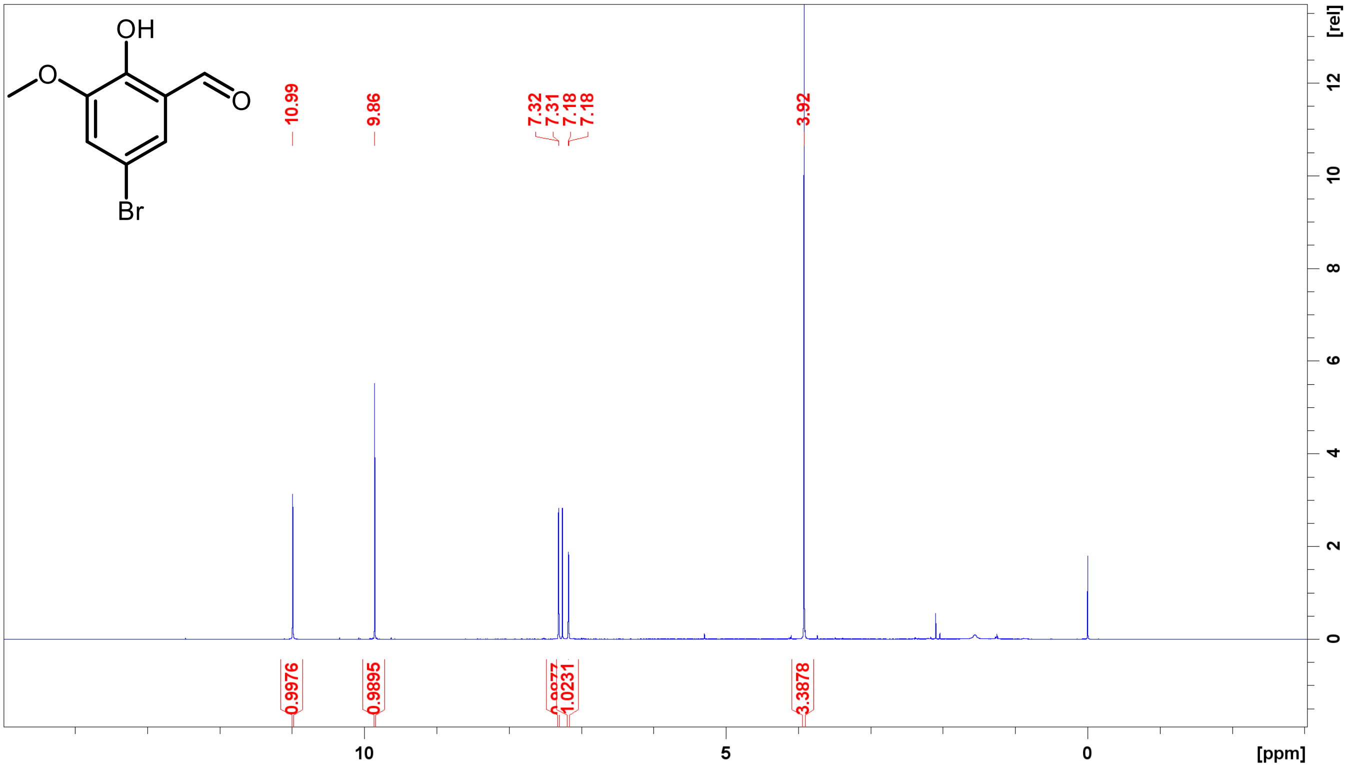

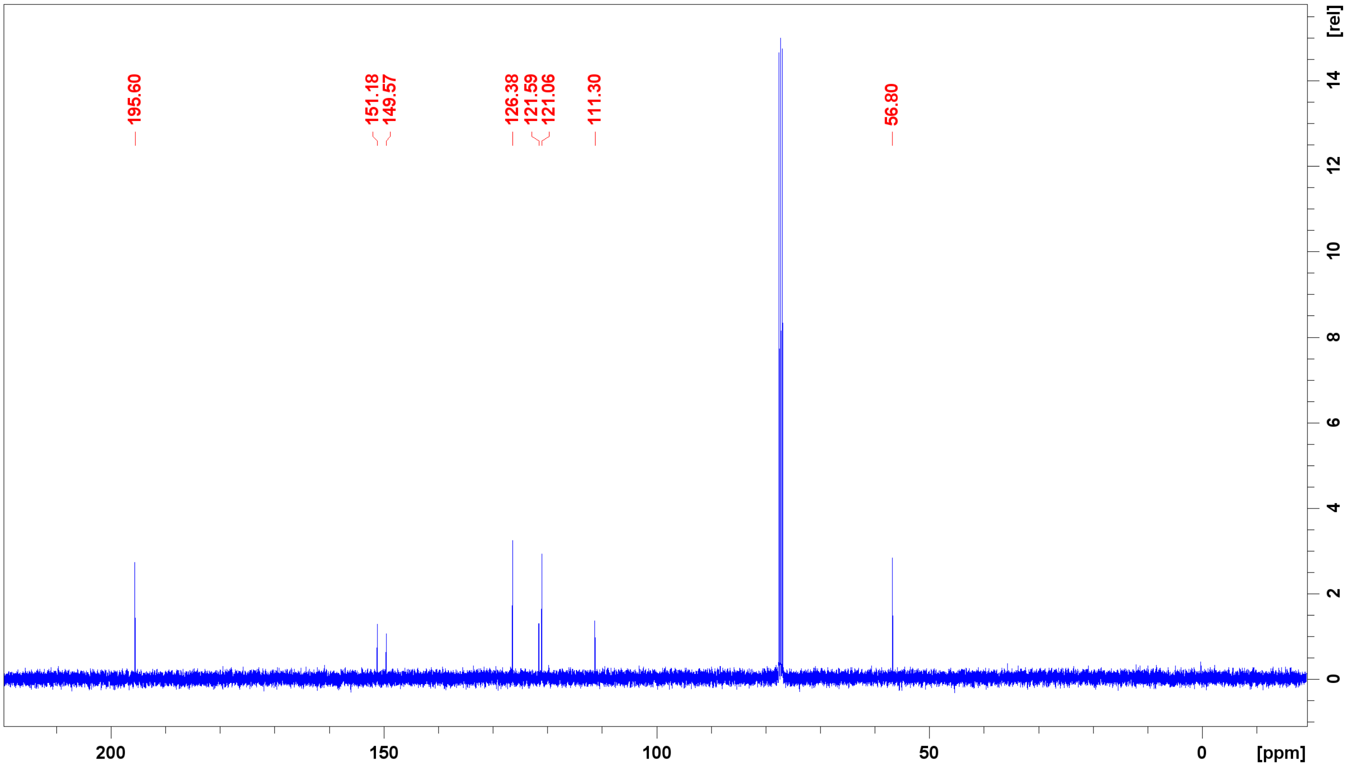

Figure S15. ^{1}H & ^{13}C spectra of compound **14**.

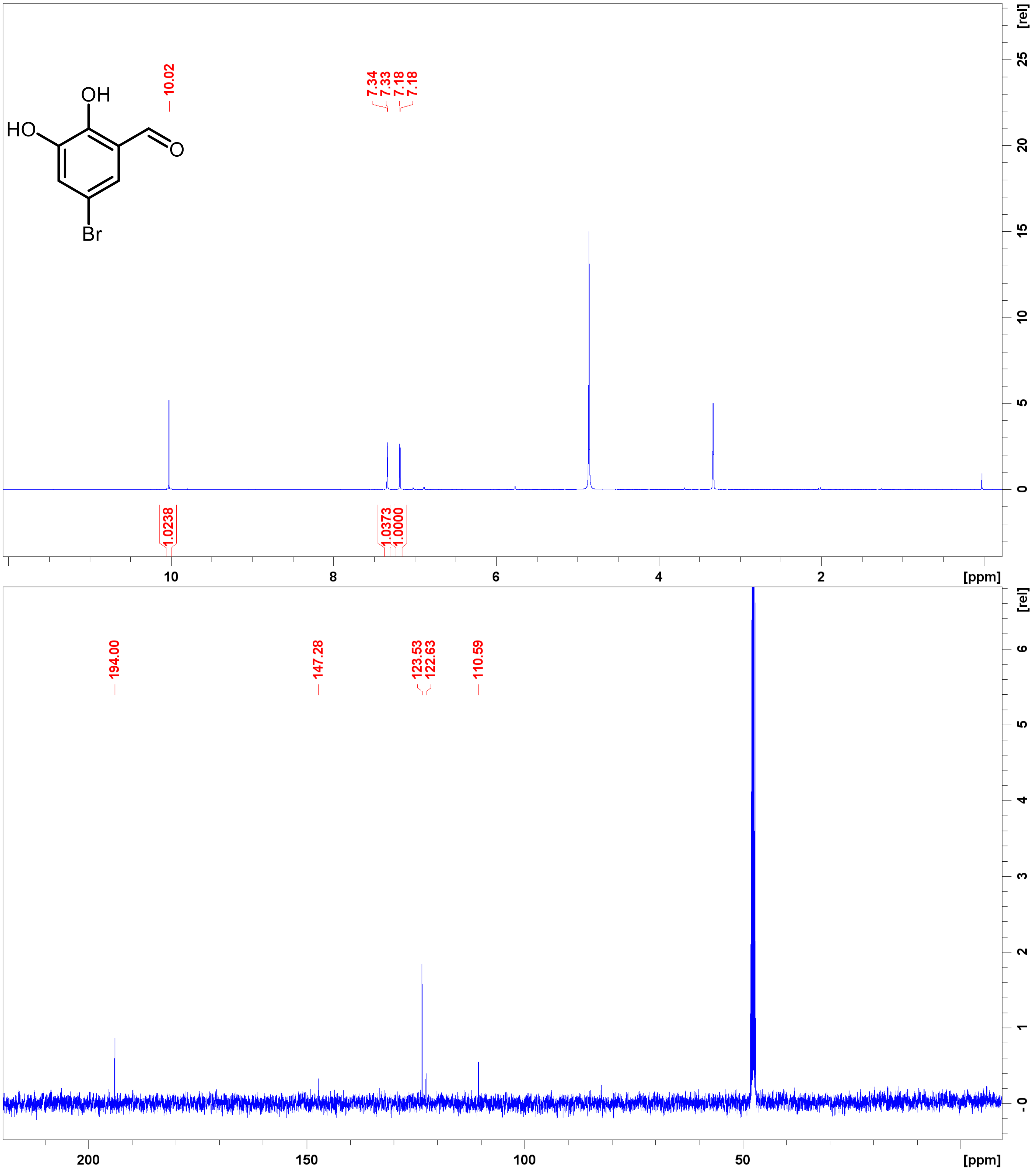

Figure S16. ^{1}H & ^{13}C spectra of compound **15**.

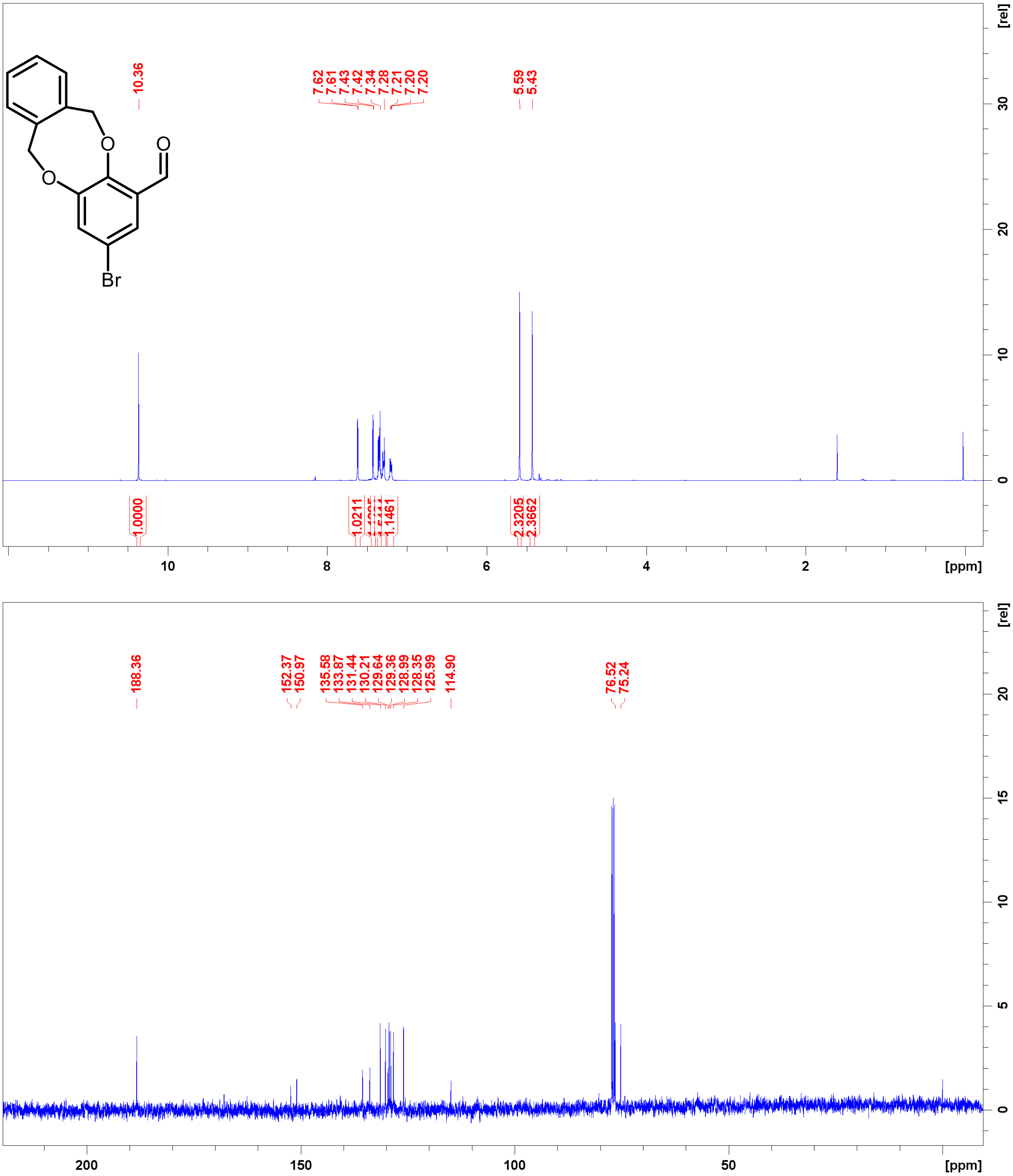

Figure S17. ^{1}H & ^{13}C spectra of compound **16**.

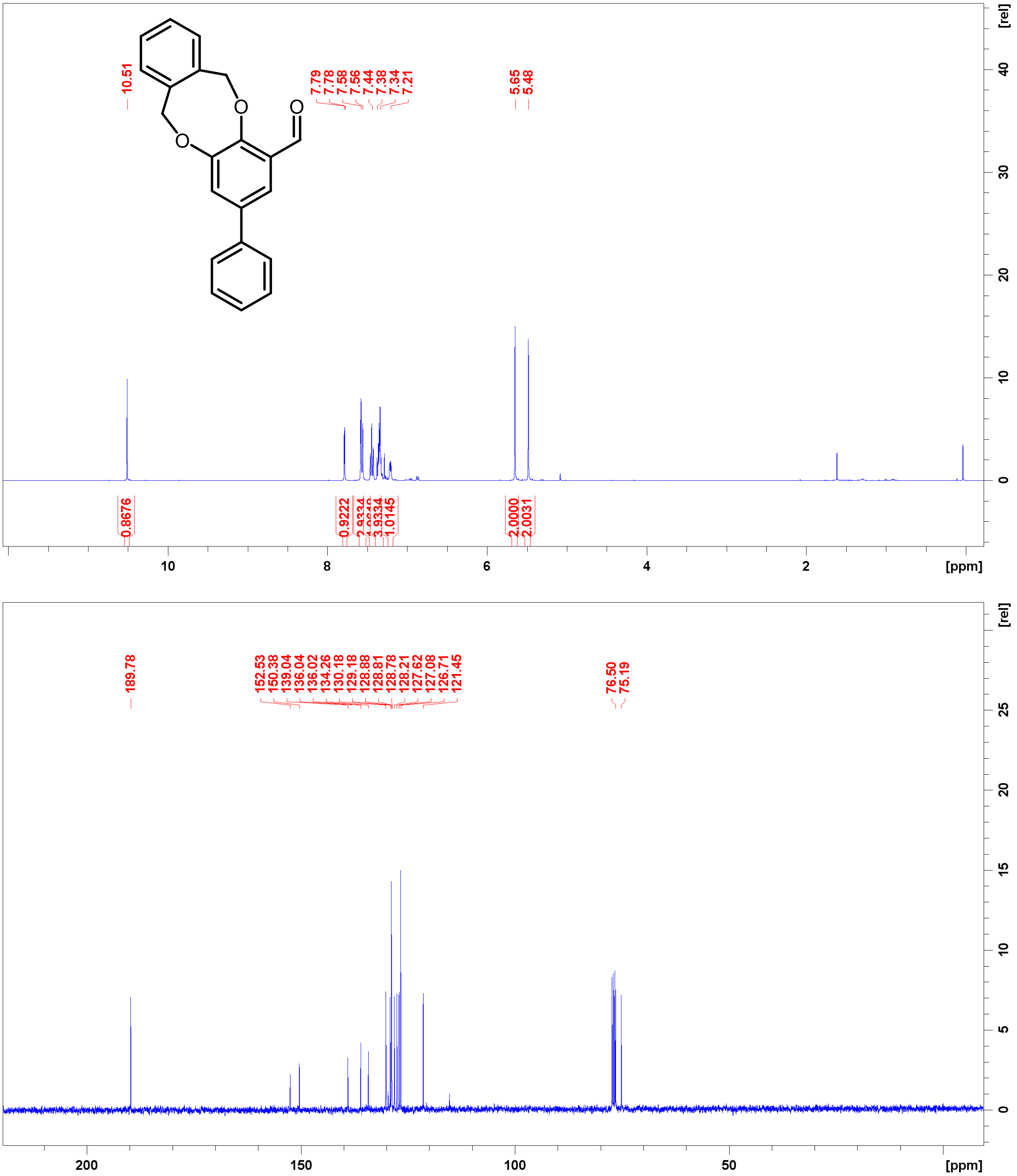

Figure S18. ^{1}H & ^{13}C spectra of compound **S7**.

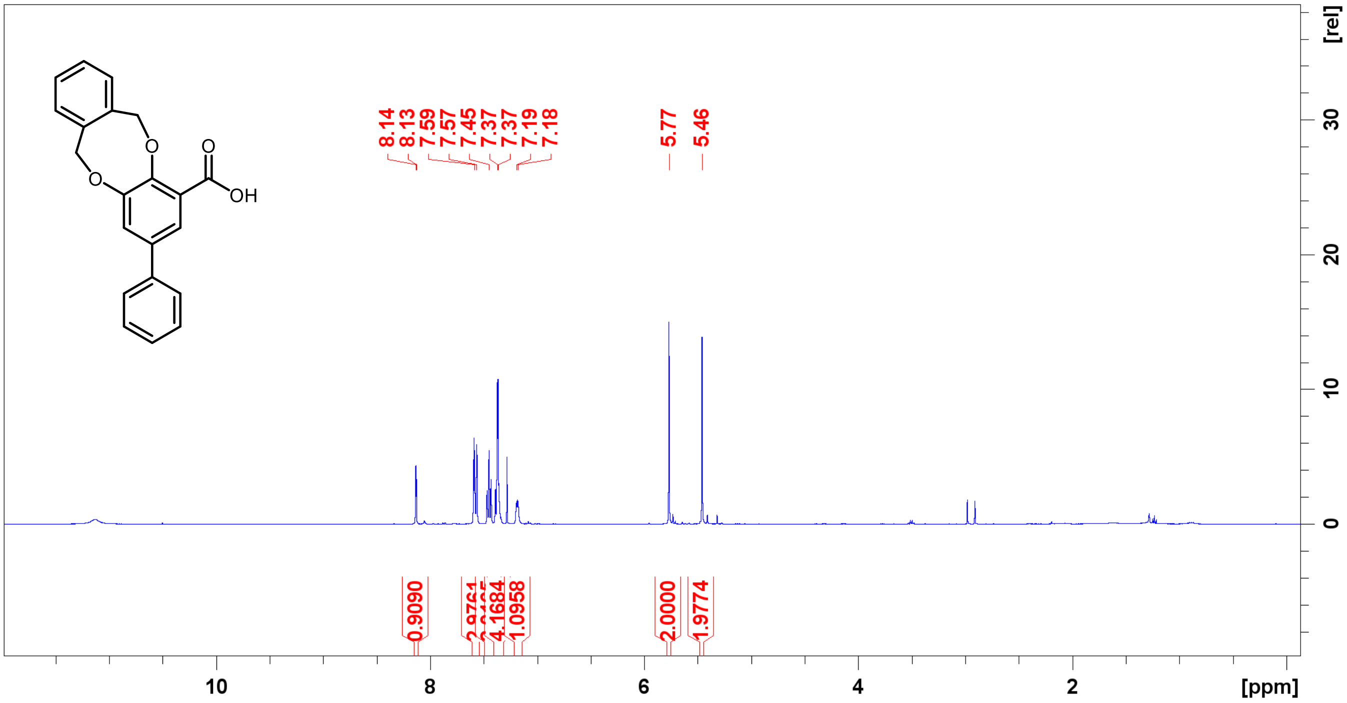

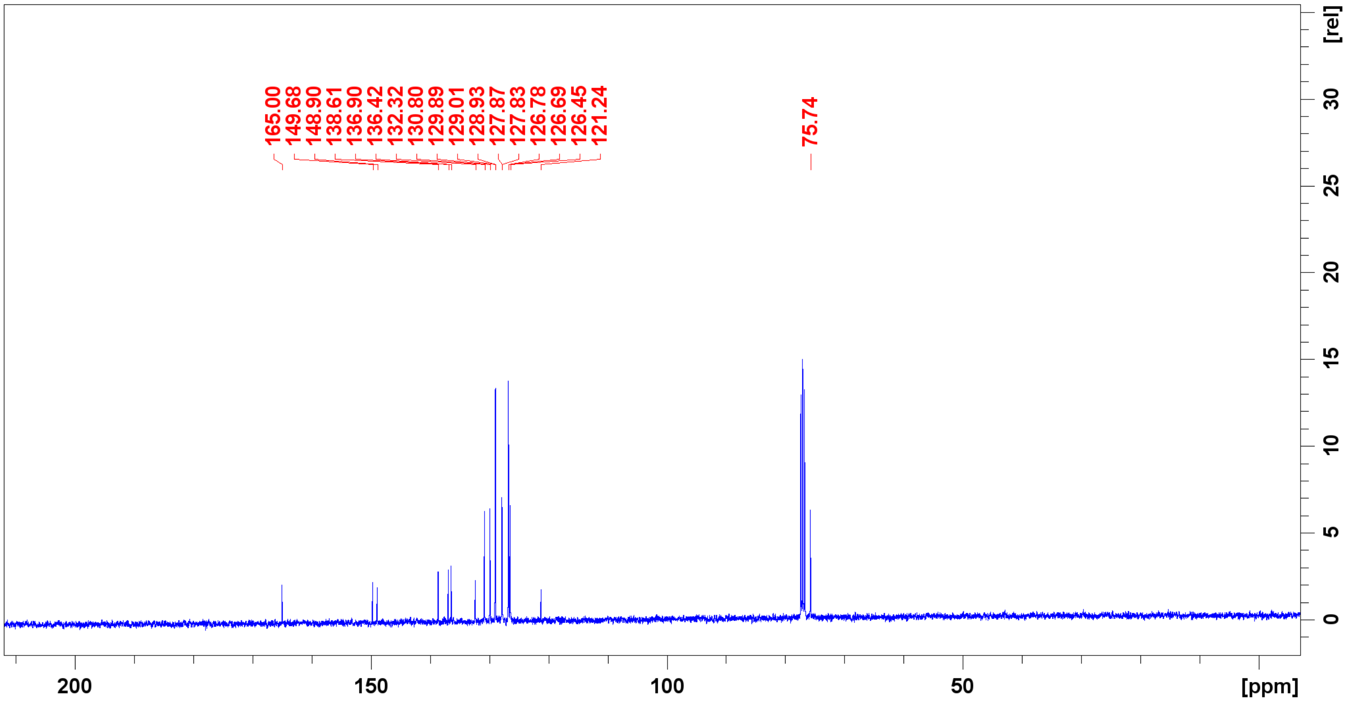

Figure S19. ^{1}H & ^{13}C spectra of compound **17**.

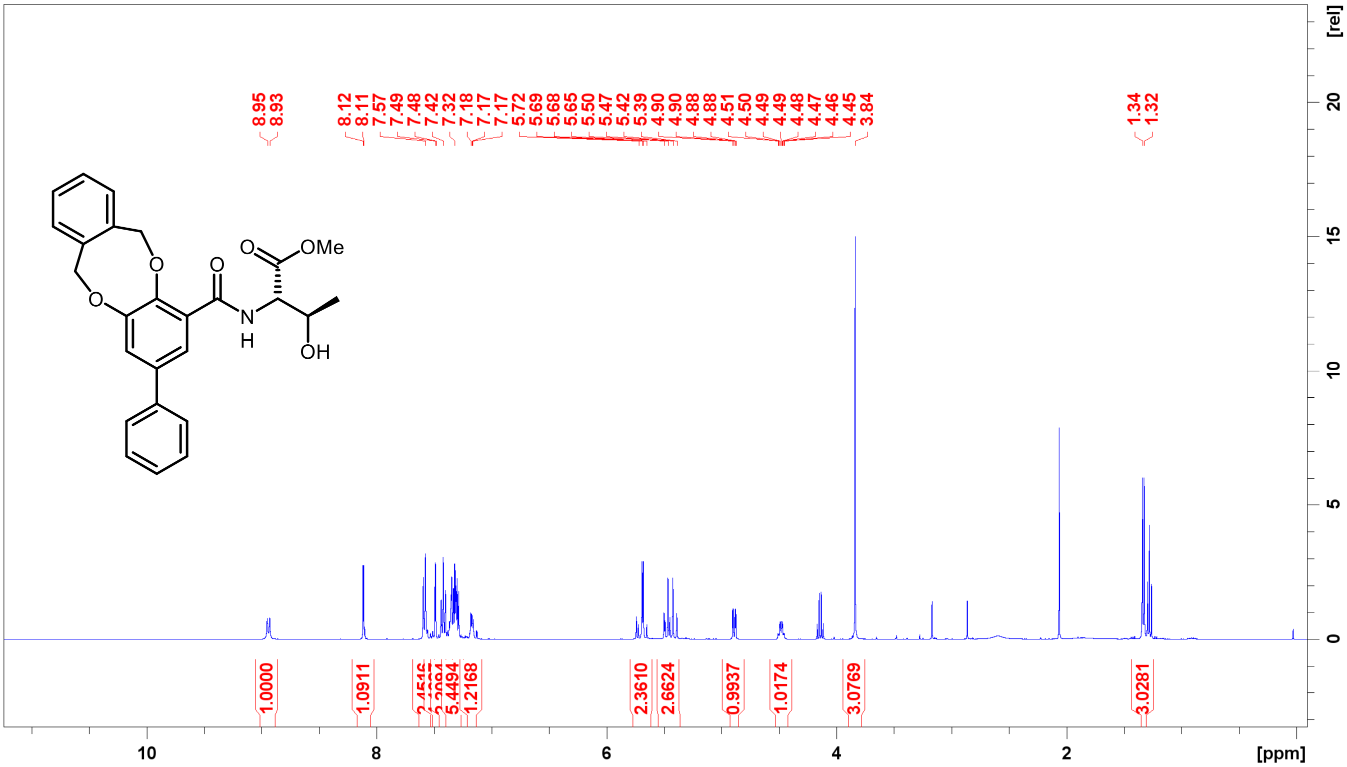

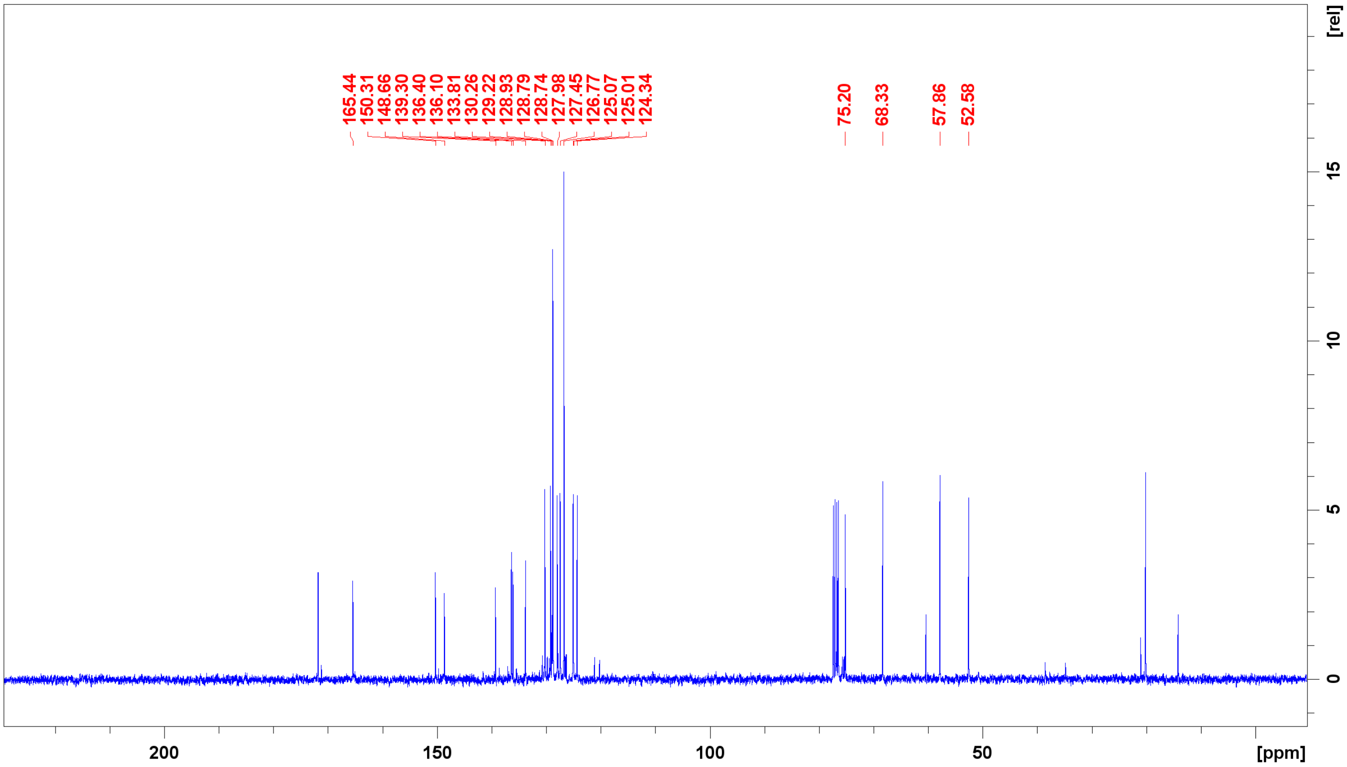

Figure S20. ^{1}H & ^{13}C spectra of compound **18**.

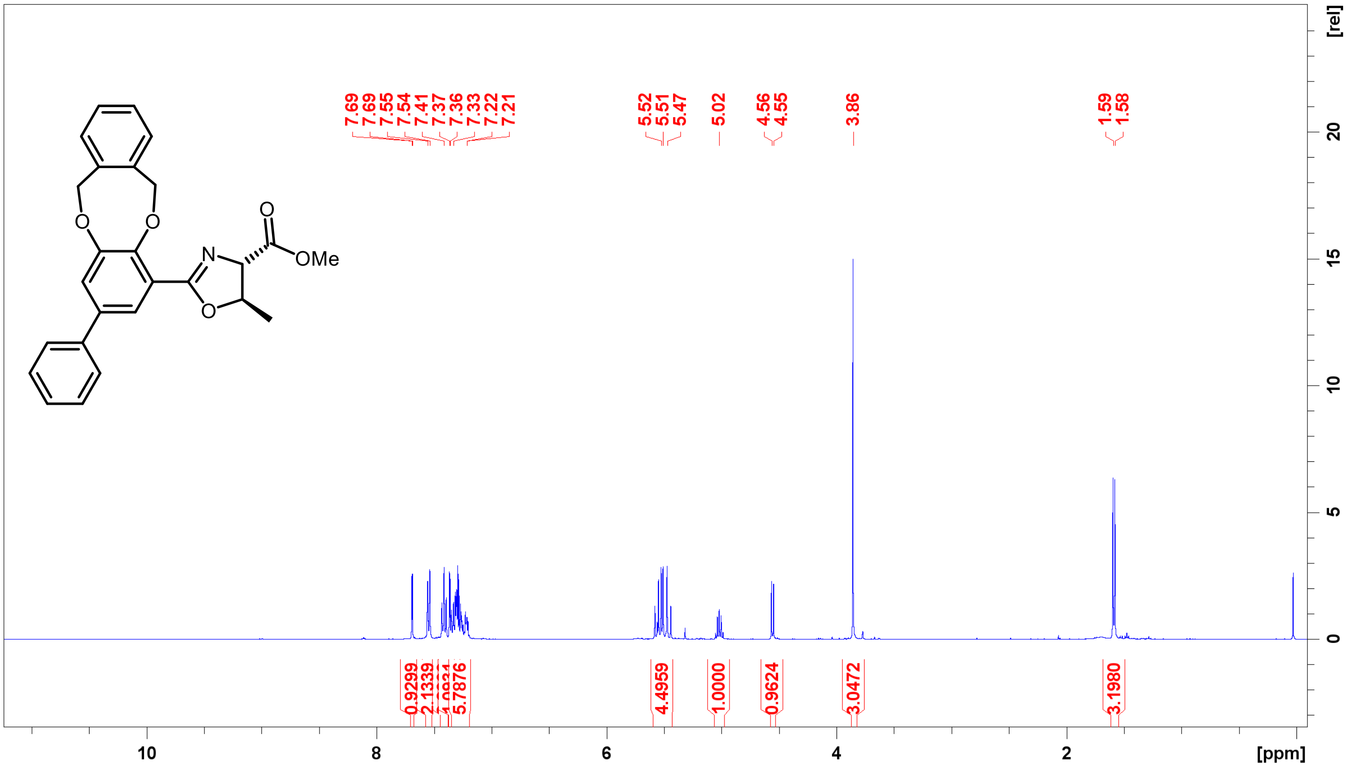

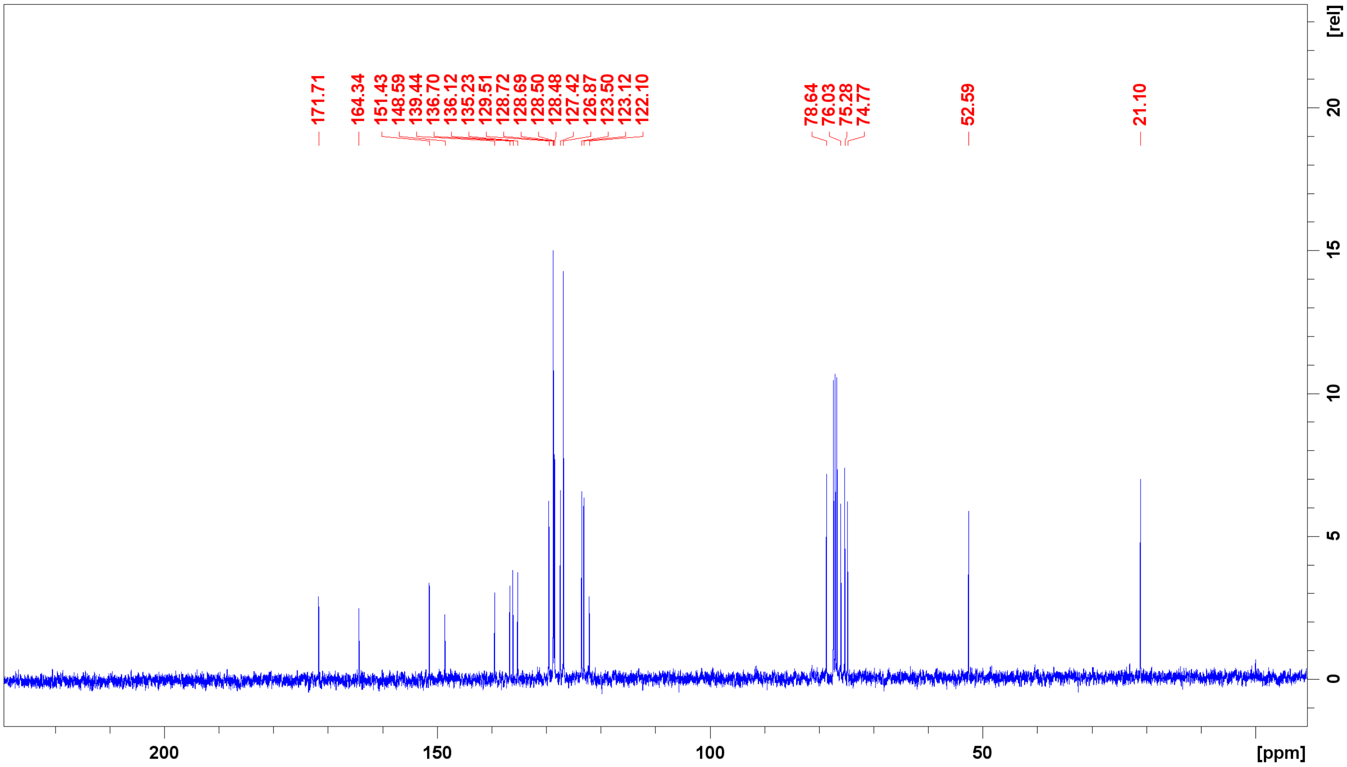

Figure S21. ^1H & ^{13}C spectra of compound **20**.

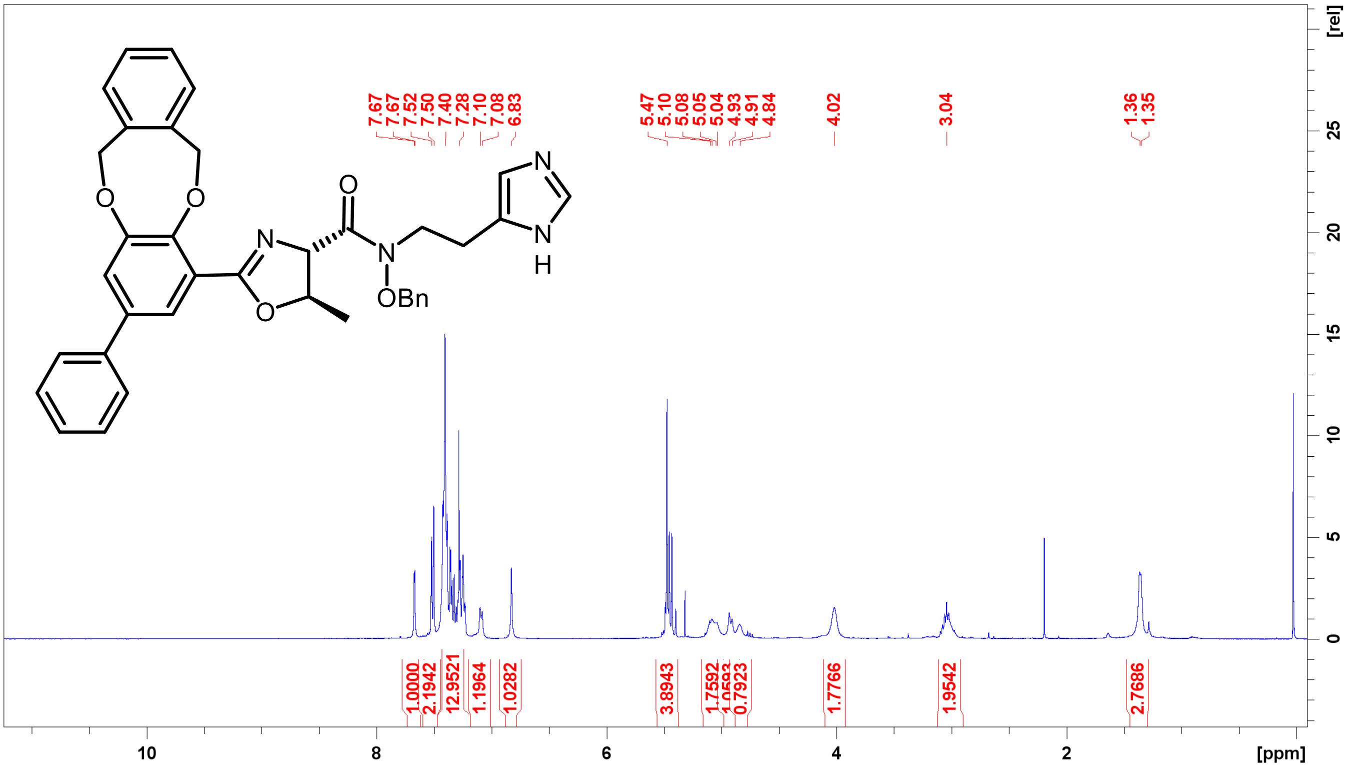

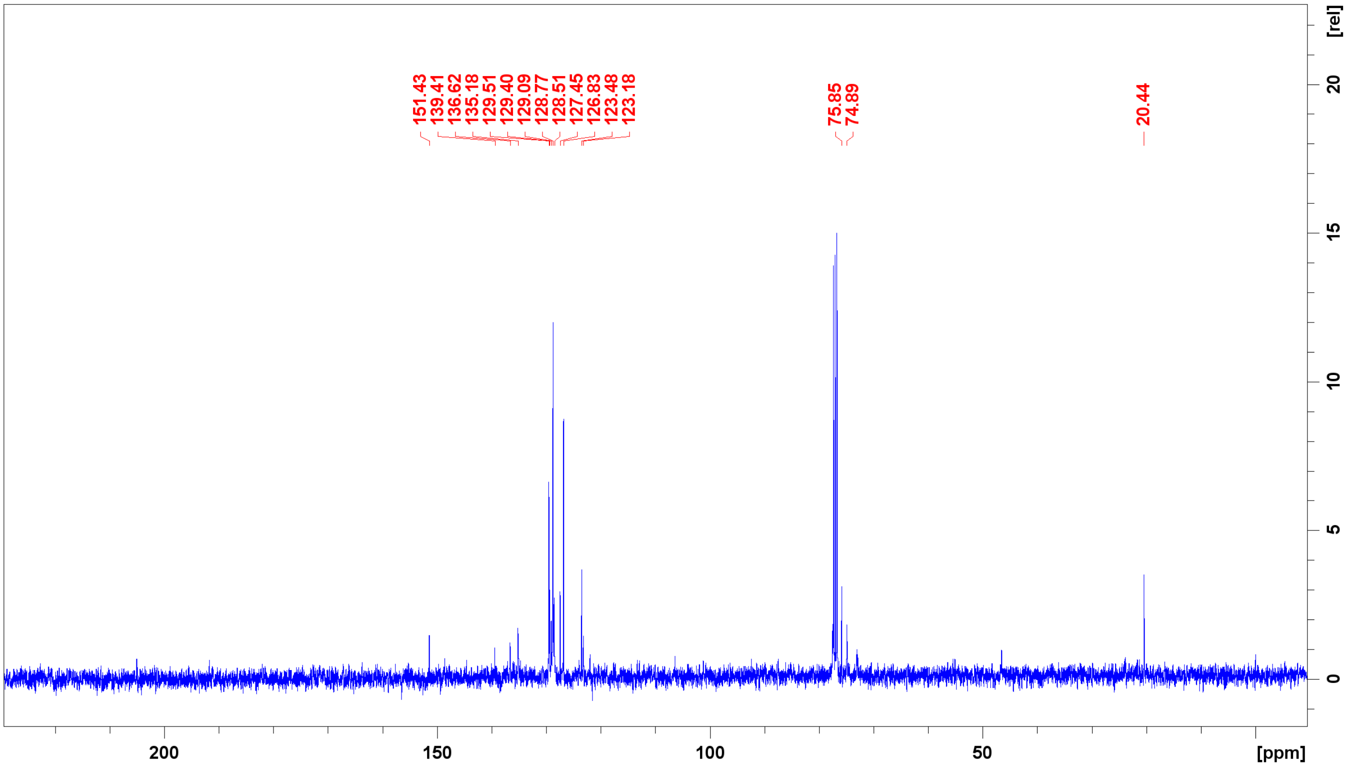

Figure S22. ^1H & ^{13}C spectra of 5-phenyl preacinetobactin **21**.

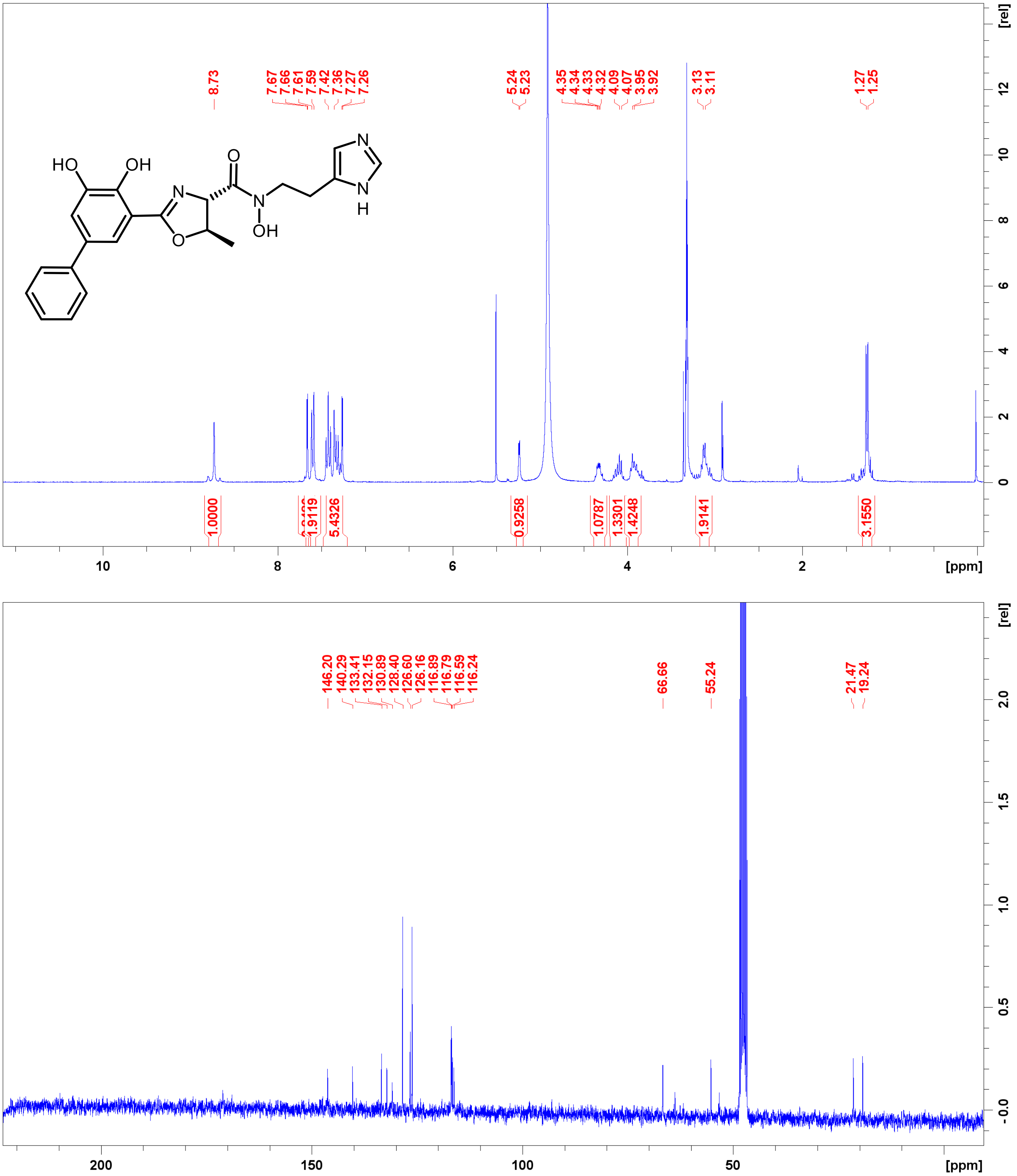

Supplement: Supplementary file 1 [file molecules-27-03688-s001.zip › molecules-1736144-supplementary.pdf]
